# Oxygen-sensitive methylation of ULK1 is required for hypoxia-induced autophagy

**DOI:** 10.1038/s41467-022-28831-6

**Published:** 2022-03-04

**Authors:** Jingyi Li, Tao Zhang, Tao Ren, Xiaoyu Liao, Yilong Hao, Je Sun Lim, Jong-Ho Lee, Mi Li, Jichun Shao, Rui Liu

**Affiliations:** 1 The Second Affiliated Hospital of Chengdu Medical College, China National Nuclear Corporation 416 Hospital, Chengdu, 610051 China; 2 School of Biological Sciences and Technology, Chengdu Medical College, Chengdu, 610599 China; 3 Oncology Department, Clinical Medical College and The First Affiliated Hospital of Chengdu Medical College, Chengdu, 610500 China; 4 State Key Laboratory of Oral Diseases, National Clinical Research Center for Oral Diseases, Chinese Academy of Medical Sciences Research Unit of Oral Carcinogenesis and Management, West China Hospital of Stomatology, Sichuan University, Chengdu, Sichuan 610041 P. R. China; 5 Stomatology Hospital, School of Stomatology, Cancer Center, Zhejiang University School of Medicine, and Key Laboratory of Oral Biomedical Research of Zhejiang Province, Clinical Research Center of Oral Diseases of Zhejiang Province, Hangzhou, 310006 Zhejiang China; 6 Department of Health Sciences, The Graduate School of Dong-A University, Busan, 49315 Republic of Korea; 7 Department of Biomedical Sciences, Dong-A University, Busan, 49315 Republic of Korea; 8 MD Anderson UTHealth Graduate School of Biomedical Sciences, Houston, 77030 TX USA; 9 Department of Experimental Radiation Oncology, The University of Texas MD Anderson Cancer Center, Houston, 77030 TX USA

**Keywords:** Macroautophagy, Stress signalling, Methylation, Autophagosomes

## Abstract

Hypoxia is a physiological stress that frequently occurs in solid tissues. Autophagy, a ubiquitous degradation/recycling system in eukaryotic cells, renders cells tolerant to multiple stressors. However, the mechanisms underlying autophagy initiation upon hypoxia remains unclear. Here we show that protein arginine methyltransferase 5 (PRMT5) catalyzes symmetrical dimethylation of the autophagy initiation protein ULK1 at arginine 170 (R170me2s), a modification removed by lysine demethylase 5C (KDM5C). Despite unchanged PRMT5-mediated methylation, low oxygen levels decrease KDM5C activity and cause accumulation of ULK1 R170me2s. Dimethylation of ULK1 promotes autophosphorylation at T180, a prerequisite for ULK1 activation, subsequently causing phosphorylation of Atg13 and Beclin 1, autophagosome formation, mitochondrial clearance and reduced oxygen consumption. Further, expression of a ULK1 R170K mutant impaired cell proliferation under hypoxia. This study identifies an oxygen-sensitive methylation of ULK1 with an important role in hypoxic stress adaptation by promoting autophagy induction.

## Abstract (web summary)

Hypoxia induces mitochondrial clearance and autophagy, although the upstream mechanisms are not well defined. Here, the authors identify that oxygen-sensitive methylation of the key autophagy regulator ULK1 promotes ULK1 activation and subsequent autophagosome formation and mitochondrial clearance.

## Introduction

Hypoxia is caused by insufficient supply of oxygen, which is a cofactor or substrate for various biological enzymatic reactions. Cells may gain tolerance in low oxygen environment, by either promoting angiogenesis to increase oxygen supply or enhancing mitochondria clearance to limit oxygen consumption^1,2^. Hypoxia-inducible factor (HIF) act as a sensor to detect cellular oxygen levels, and binds to the hypoxia-responsive elements under hypoxia condition, thereby initiating the transcription of its target genes^3^. Oxygen supply in rapid growing tumors is frequently impaired as a result of disturbed microcirculation, inevitably leading to hypoxic stress in tumor cells^4^.

Autophagy is one of the cellular adaptive responses under multiple stresses, which enables intracellular materials delivered to lysosomes for degradation, and the products can be subsequently exported to the cytoplasm to be recycled^5^. As a highly conserved process in all eukaryotes, autophagy plays important roles in regulating cellular metabolism as well as tissue homeostasis by removing damaged macromolecules or even organelles^6,7^. On the other hand, abnormal autophagy is closely associated with human diseases, including cancers^8–11^.

Autophagy is regulated by a complex molecular network. Atg1/Unc-51 like autophagy activating kinase 1 (ULK1) is a key regulator of autophagy initiation^12^. At early steps during autophagy induction, active ULK1 regulates the recruitment of Atg14L-containing vacuolar sorting protein 34 (VPS34) complex^13,14^. ULK1 then phosphorylates Beclin 1, a binding partner for VPS34, thereby enhancing the activity of VPS34 complexes^15–17^. VPS34, the only class III phosphoinositide 3-kinase (PI3K) in mammals, phosphorylates phosphatidylinositol to produce phosphatidylinositol 3-phosphate (PI3P), which is necessary for phagophore membrane extension^18^. Finally, two ubiquitin-like conjugation systems promote lipidation of Microtubule-Associated Protein 1 Light Chain 3 (LC3), leading to cargo recognition and packaging in autophagosomes^19–21^. Though HIF1α plays an important role in autophagy induction by releasing Beclin 1 from Bcl-2^22,23^, the mechanisms underlying autophagy initiation under hypoxic stress remains unclear.

In this study, we demonstrated that symmetrical dimethylation of ULK1 arginine (R)170, which is governed by PRMT5 and KDM5C, is sensitive to oxygen availability. This modification of ULK1 promotes autophagosome formation and mitochondria clearance, and is required for cell adaptation to hypoxic stress.

## Results

### Hypoxia-induced symmetrical dimethylation at ULK1 R170 is required for autophagy induction

**Fig. 1:**
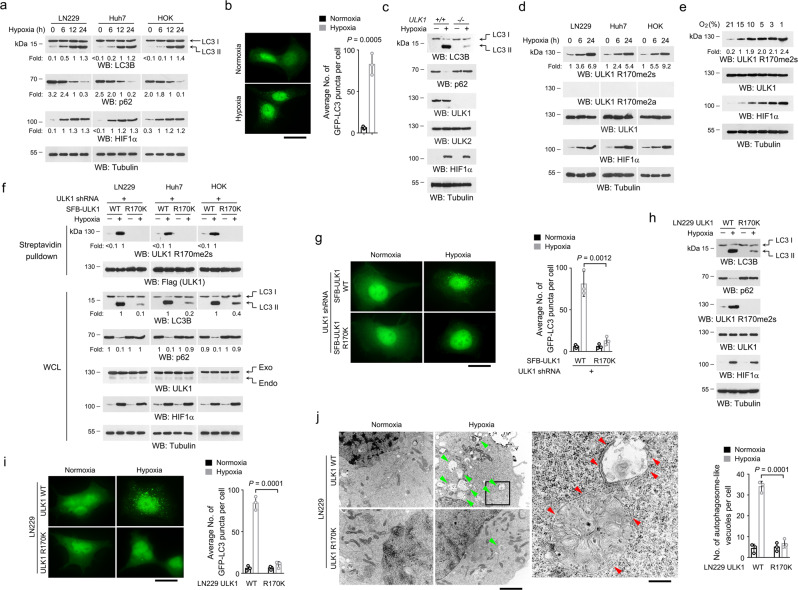
ULK1 R170me2s is required for hypoxia-induced autophagy. **a**, **c**–**f**, **h** Immunoblot were performed with the indicated antibodies. **a** LN229, Huh7, and HOK cells were incubated with 1% oxygen. **b** LN229 cells expressed with LC3–GFP were incubated with 1% oxygen for 12 h. LC3–GFP puncta were analyzed. Scale bar, 15 µm. Data represent the mean ± SD from three independent experiments. *P*-value is from the two-sided *t*-test. **c** WT and ULK1−/− MEFs were incubated with 1% oxygen for 12 h. **d** LN229, Huh7, and HOK cells were incubated with 1% oxygen. **e** LN229 cells were incubated with indicated concentration of oxygen for 12 h. **f** Endogenous ULK1-depleted LN229, Huh7, and HOK cells with reconstituted expression of WT SFB-ULK1 or SFB-ULK1 R170K were incubated with 1% oxygen for 12 h. A streptavidin pulldown was performed. ULK1 shRNA targets non-coding region. Exo, exogenous; Endo, endogenous. **g** Endogenous ULK1-depleted LN229 cells with reconstituted expression of SFB-tagged WT ULK1 or SFB-ULK1 R170K were transfected with LC3–GFP plasmid. 48 h after transfection, cells were incubated with 1% oxygen for 12 h. LC3–GFP puncta were analyzed. Scale bar, 10 µm. ULK1 shRNA targets non-coding region. Data represent the mean ± SD from three independent experiments. *P*-value is from the two-sided *t*-test. **h**, **j** Indicated cells were incubated with 1% oxygen for 12 h. Immunoblot were performed (**h**). Ultrastructure was analyzed by TEM (**j**). The boxed area was enlarged. Green arrowheads indicate autophagosome-like vacuoles, and red arrowheads indicate double membrane structures. Scale bar for original images, 2 µm; scale bar for enlarged images, 400 nm. Data represent the mean ± SD from three independent experiments. *P*-value is from the two-sided *t*-test. **i** WT LN229 cells and LN229 cells with knockin expression of ULK1 R170K were transfected with LC3–GFP plasmid. 48 h after transfection, cells were incubated with 1% oxygen for 12 h. LC3–GFP puncta were analyzed. Data represent the mean ± SD from three independent experiments. *P*-value is from the two-sided *t*-test. Scale bar, 10 µm. Source data are provided as a Source data file.

To examine the effect of hypoxia on autophagy induction, we examined the levels of autophagy markers, LC3 II and p62, in LN229 human glioblastoma (GBM) cells, Huh7 hepatocellular carcinoma (HCC) cells, and human oral keratinocytes (HOKs) cultured in the hypoxic environment (1% oxygen). In all three cell lines, a dynamically increased LC3 II expression companied with a reduced p62 expression was detected (Fig. 1a). In addition, hypoxia resulted in increased puncta formation of LC3–GFP fusion protein in LN229 cells (Fig. 1b). These observations indicated that autophagy was efficiently activated in these cell lines. Hypoxia represses mTOR and activates AMPK, which are known as key upstream regulators of autophagy^24,25^. Deficiency of TSC1, which resulted in constant active mTOR and enhanced phosphorylation of mTOR substrate p70 Ribosomal S6 Kinase (S6k), or double knockout of AMPKα1α2, which caused declined phosphorylation of AMPK substrate ACC, did not affect hypoxia-induced accumulation of LC3 II or decrease of p62 (Supplementary Fig. 1a, b), suggesting that hypoxia-induced autophagy is independent of mTOR or AMPK.

ULK1 and ULK2 are pivotal players in autophagy initiation^12^. Knockout of ULK1 in mouse embryo fibroblasts (MEFs) reduced hypoxia-induced LC3 II accumulation and restored p62 expression (Fig. 1c), while no obvious effects were observed in ULK2 knockout MEFs (Supplementary Fig. 1c). This is consistent with previous reports that, in spite of some shared functions, ULK1 and ULK2 have distinct roles in autophagy induction^22,26^. AMPK and mTOR regulate autophagy through phosphorylating ULK1^22^. However, a time-course experiment showed no obvious change in either mTOR-induced phosphorylation of ULK1 S757 or AMPK-mediated phosphorylation of ULK1 S555 under 12 h-hypoxia treatment (Supplementary Fig. 1d), which could efficiently induce autophagy (Fig. 1a). ULK1 immunoprecipitates derived from hypoxia-stimulated LN229 cells was subjected to liquid chromatography-tandem mass spectrometry, leading to identification of a dimethylation at the evolutionally conserved ULK1 R170 (Supplementary Fig. 1e, f). Though a canonical ‘RGG’ motif is reported, arginine methylations were also found at various non-canonical sites^27,28^. Hypoxia-induced ULK1 R170 dimethylation was detected with a validated anti-symmetrically dimethylated R170 (R170me2s) antibody, but not anti-asymmetrically dimethylated R170 (R170me2a) antibody, in LN229, Huh7 or HOK cells (Fig. 1d, Supplementary Fig. 1g). The immunoblot signal could be abolished by incubation with a R170me2s, but not R170me, R170me2a or unmodified peptide (Supplementary Fig. 1h). Further, ULK1 R170me2s showed a dynamic response to oxygen availability in LN229 cells, and this modification was markedly increased when oxygen tension was decreased below 10% (Fig. 1e). Though ULK1 and ULK2 share comparable protein molecular weight and similar protein sequence spanning ULK1 R170 and the corresponding ULK2 R163 (Supplementary Fig. 1i), the anti-ULK1 R170me2s antibody recognized immunoprecipitated Flag-ULK1, rather than Flag-ULK2, from hypoxia-stimulated LN229 cells (Supplementary Fig. 1j). Consistently, only ULK1 deficiency totally blocked hypoxia-induced immunoblot signal using the anti-ULK1 R170me2s antibody (Supplementary Fig. 1k). These results suggested that hypoxic stress induces ULK1 R170me2s, while ULK2 was not methylated at the corresponding site R163.

To determine the effect of ULK1 R170me2s on hypoxia-induced autophagy, we depleted endogenous ULK1 expression by short hairpin RNA (shRNA), and established the reconstituted expression of triple-epitope S protein/FLAG/streptavidin-binding peptide (SFB)-tagged ULK1 R170lysine (K) mutant, which resists R170me2s, in LN229, Huh7 and HOK cells. Indeed, ULK1 R170K mutation substantially reduced hypoxia-induced accumulation of LC3 II, clearance of p62 (Fig. 1f) and formation of LC3–GFP puncta (Fig. 1g). Additionally, though loss of TSC1 or AMPKα1α2 showed negligible effects on the level of ULK1 R170me2s (Supplementary Fig. 1l, m), reconstituted expression of ULK1 R170K mutant largely attenuated autophagy induction in TSC1 or AMPKα1α2 knockout MEFs (Supplementary Fig. 1n, o). Further, we employed CRISPR/Cas9 genome-editing technology to knockin ULK1 R170K mutation in LN229 cells (Supplementary Fig. 2a). As expected, knockin expression of ULK1 R170K impeded autophagy induction in hypoxia-stimulated LN229 cells, as detected by immunoblot analyses of LC3 II and p62 expression, fluorescent analyses of LC3–GFP puncta and transmission electron microscope (TEM) analyses of the double-membraned autophagosome-like vacuoles (Fig. 1h–j). Further, treatment of chloroquine (CQ) or analyses of LC3-RFP-GFP puncta revealed that ULK1 R170K mutation also blocked hypoxia-induced autophagic flux (Supplementary Fig. 2b–d). In contrast, ULK1 R170me2s was not obviously changed upon glucose or amino acids starvation, and ULK1 R170K mutation showed limited effects on autophagy induction in response to these stimuli (Supplementary Fig. 2e, f). These results strongly suggest that hypoxia-induced ULK1 R170me2s activates autophagy in an mTOR or AMPK-independent manner.

### ULK1 R170 is symmetrically dimethylated by PRMT5 and demethylated by KDM5C

**Fig. 2:**
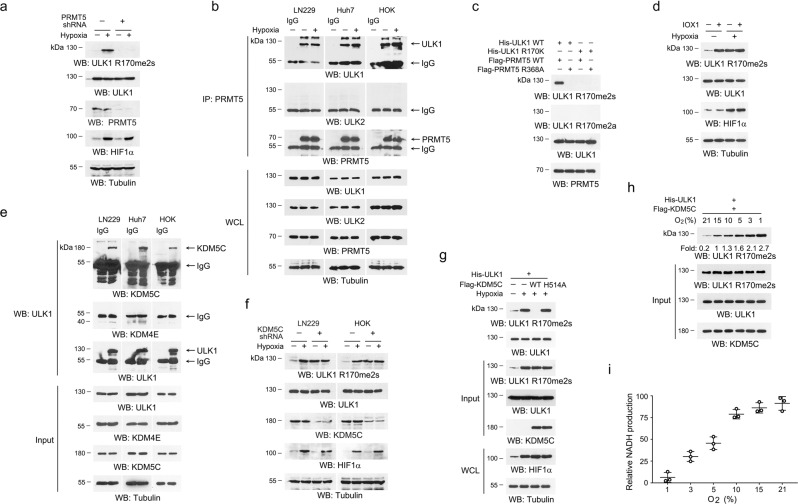
ULK1 R170 is symmetrically dimethylated by PRMT5 and demethylated by KDM5C. **a**–**h** Immunoblot were performed with the indicated antibodies. **a** LN229 cells with or without expression of PRMT5 shRNA were incubated with 1% oxygen for 12 h. **b** LN229, Huh7 or HOK cells were incubated with 1% oxygen for 12 h. Immunoprecipitations were performed using an anti-PRMT5 antibody. **c** Purified WT His-ULK1 or His-ULK1 R170K protein was mixed with purified WT Flag-PRMT5 or Flag-PRMT5 R368A protein for an in vitro methylation assay. **d** LN229 cells were pretreated with or without 200 mM IOX1 for 12 h, followed by incubation under 1% oxygen for 12 h. **e** LN229, Huh7 or HOK cells were cultured in normoxia condition. Immunoprecipitations were performed using an anti-ULK1 antibody. **f** LN229 and HOK cells with or without expression of KDM5C shRNA were incubated with 1% oxygen for 12 h. **g** LN229 with expression of His-ULK1 were incubated under 1% oxygen for 12 h. His-ULK1 proteins were pulldown, the precipitates were mixed with purified WT Flag-KDM5C or Flag-KDM5C H514A mutant protein for an in vitro demethylation assay in normoxia condition. **h** LN229 with expression of His-ULK1 were incubated under 1% oxygen for 12 h. His-ULK1 proteins were pulldown, washed and mixed with purified WT Flag-KDM5C protein for an in vitro demethylation assay in presence of indicated concentrations of oxygen. **i** Purified Flag-KDM5C protein was mixed with ULK1 R170me2s peptide for an in vitro demethylation assay in presence of indicated concentrations of oxygen. Data represent the mean ± SD from three independent experiments. Source data are provided as a Source data file.

Generation of asymmetric dimethylarginines is catalyzed by type I protein arginine methyltransferases (PRMTs), while type II enzymes, PRTM5 and PRMT7, catalyzes symmetric arginine dimethylation^29^. ShRNA-mediated knockdown of PRMT5 or PRMT7 revealed that PRMT5, rather than PRMT7, was involved in ULK1 R170me2s (Fig. 2a, Supplementary Fig. 3a). As expected, association between endogenous ULK1 and PRMT5 was detected in LN229, Huh7 and HOK cells cultured in both normoxia or hypoxia conditions (Fig. 2b); in contrast, ULK2 was not detected in PRMT5 immunoprecipitates (Fig. 2b). We next conducted an in vitro methylation analysis by incubating purified WT Flag-PRMT5 or catalytic-dead Flag-PRMT5 R368A mutant^30^ protein with WT His-ULK1 or His-ULK1 R170K protein, in the presence of S-adenosylmethionine as a methyl group donor. We found that WT PRMT5, but not PRMT5 R368A mutant, symmetrically dimethylated His-ULK1, which could be blocked by R170K mutation (Fig. 2c).

We next asked whether hypoxia enhanced PRMT5-mediated ULK1 R170me2s. Nevertheless, interaction between PRMT5 and ULK1 was not altered in hypoxia-treated LN229 cells (Supplementary Fig. 3b). An in vitro methylation analysis showed that oxygen concentration was not a determinant factor for PRMT5 to methylate ULK1 R170 (Supplementary Fig. 3c). Further, depletion of HIF1α had no obvious effect on hypoxia-mediated ULK1 R170me2s (Supplementary Fig. 3d). These results suggested that, though PRMT5 catalyzes ULK1 R170me2s, hypoxia-induced ULK1 R170me2s was not caused by PRMT5 alteration and was independent of HIFα.

To find out whether hypoxia-induced ULK1 R170me2s was governed by a demethylase, LN229 cells were treated with 5-carboxy-8-hydroxyquinoline (IOX1), a pan-inhibitor of the 2-oxoglutarate-dependent JMJD family demethylase^31^. Strikingly, though IOX1 markedly enhanced ULK1 R170me2s under normoxia condition, hypoxia treatment could not further increase ULK1 R170me2s (Fig. 2d), suggesting that a demethylase was probably involved. KDM4E and KDM5C, belonging to the KDM4 and KDM5 lysine demethylase subfamilies, were recently evidenced to be capable to catalyze de-dimethylation of arginine on histone peptides (Supplementary Fig. 3e)^32^. Notably, interaction between endogenous ULK1 and KDM5C, but not KDM4E, was confirmed by immunoprecipitation in LN229, Huh7 and HOK cells (Fig. 2e).

To determine whether KDM5C was capable to regulates ULK1 R170me2s, we knockdown the expression of KDM5C by two distinct shRNAs, and found a markedly enhanced ULK1 R170me2s and autophagy induction in normoxia condition (Supplementary Fig. 3f–h). However, this effect was not further augmented by hypoxia stimulation (Fig. 2f). In contrast, overexpression of exogenous KDM5C only limitedly reduced ULK1 R170me2s in hypoxia condition (Supplementary Fig. 3i). Further, we mixed His-tagged ULK1 protein purified from hypoxia-stimulated LN229 cells with purified WT Flag-KDM5C protein or catalytic-dead Flag-KDM5C H514A protein^33^, and found that only WT Flag-KDM5C could remove ULK1 R170me2s (Fig. 2g).

To decipher how hypoxia affects KDM5C-dependent demethylation of ULK1 R170me2s, we initially tested whether hypoxia regulates KDM5C expression. However, only a slight increase in KDM5C expression could be detected in LN229, Huh7 and HOK cells after 24 h hypoxia treatment (Supplementary Fig. 3j), while the expression of PRMT5 and ULK1 was almost unchanged (Supplementary Fig. 3j). In addition, nickle-nitrilotriacetic acid-aided (Ni-NTA) pull down assay demonstrated that equal amount of KDM5C protein could be pulled down by His-tagged ULK1 under normoxia and hypoxia conditions (Supplementary Fig. 3k). KDM5C catalyzes demethylation through an oxidative reaction that requires α-ketoglutarate (αKG) and oxygen as cofactors^34^. Treatment with various dosages of membrane-permeable dimethyl (DM)-αKG did not alter ULK1 R170me2s under hypoxia (Supplementary Fig. 3l), suggesting that hypoxia-regulated KDM5C is not due to αKG deficiency. To determine whether KDM5C activity was regulated by oxygen availability, purified KDM5C protein was mixed with R170-dimethylated ULK1 protein, purified from hypoxia-stimulated LN229 cells, for an in vitro demethylation assay under different oxygen concentrations. We found that ULK1 R170me2s was dynamically increased as oxygen concentration decreased (Fig. 2h). Further, impeded KDM5C activity was also observed by using the ULK1 R170me2s peptide as a substrate (Fig. 2i); a rapid change of KDM5C activity was found when oxygen tension was reduced below 10% (Fig. 2i), which was consistent with cellular ULK1 R170me2s status (Fig. 1e). These results suggested that KDM5C activity is sensitive to oxygen availability, and hypoxia-induced ULK1 R170me2s is due to repressed KDM5C-mediated demethylation.

### R170me2s activates ULK1 by facilitating ULK1 T180 autophosphorylation

**Fig. 3:**
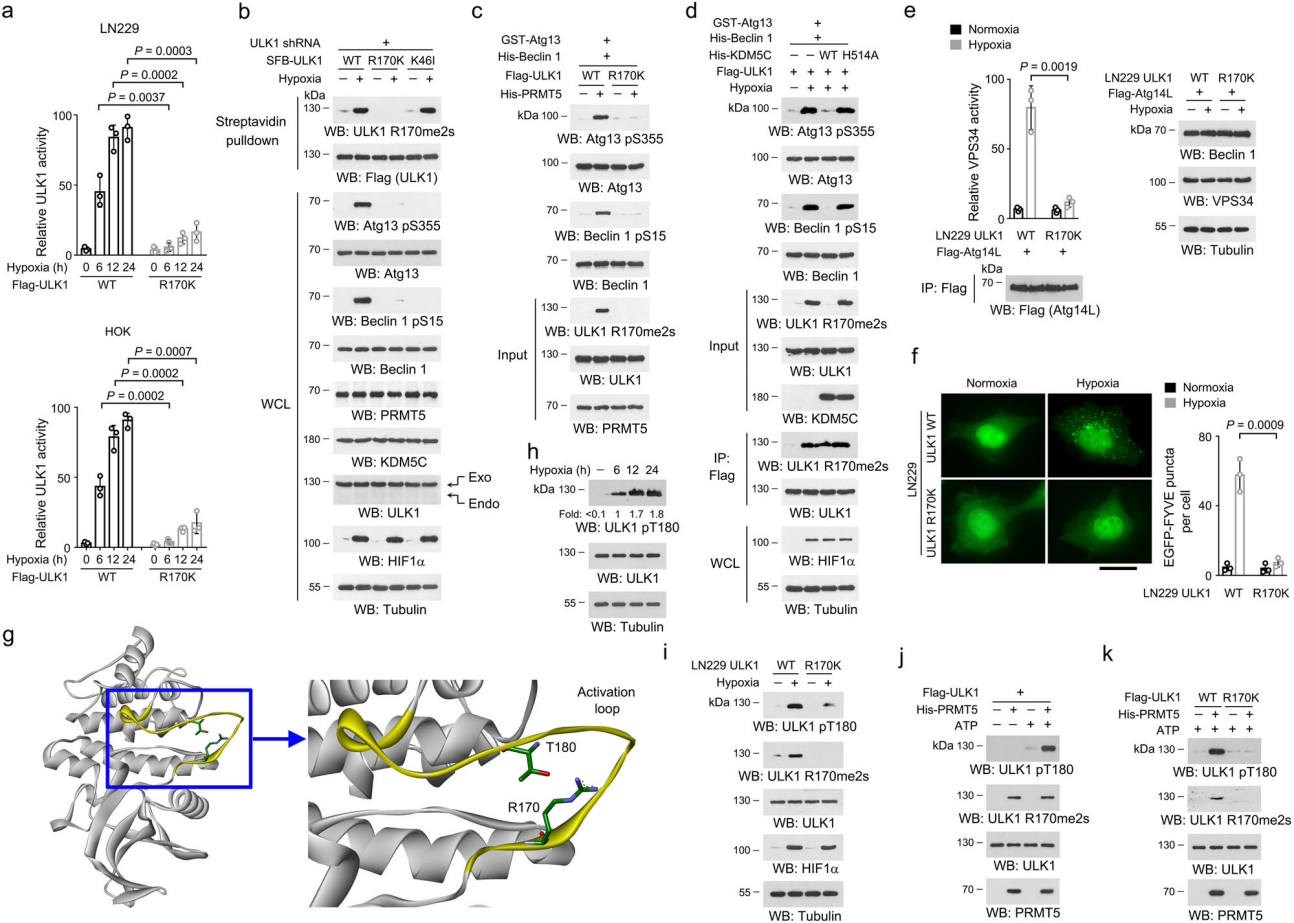
R170me2s activates ULK1 by facilitating ULK1 T180 autophosphorylation. **b**–**e**, **h**–**k** Immunoblot were performed with the indicated antibodies. **a** Indicated cells were incubated under 1% oxygen for 12 h. Flag-ULK1 proteins were precipitated, and ULK1 kinase activity was analyzed. Data represent the mean ± SD from three independent experiments. *P*-values are from the two-sided *t*-tests. Bonferroni correction was used for multiple hypothesis correction. **b** Indicated LN229 cells incubated under 1% oxygen for 12 h. Exo, exogenous; Endo, endogenous. **c** Purified ULK1 proteins was immobilized on beads, and mixed with purified His-PRMT5 protein for an in vitro methylation assay. Immobilized ULK1 proteins were washed, and mixed with purified GST-Atg13 or His-Beclin 1 proteins for an in vitro kinase assay. **d** LN229 cells with expression of Flag-ULK1 were incubated under 1% oxygen for 12 h. Flag-ULK1 proteins were pulldown, washed and mixed with purified WT His-KDM5C or His-KDM5C H514A protein for an in vitro demethylation assay under normoxia condition. The ULK1 precipitates were washed, and mixed with purified GST-Atg13 or His-Beclin 1 proteins for an in vitro kinase assay. **e** Indicated cells were incubated under 1% oxygen for 12 h. VPS34 activity in the precipitates were measured. Data represent the mean ± SD from three independent experiments. *P*-value is from the two-sided *t*-test. **f** Indicated cells were incubated under 1% oxygen for 12 h. EGFP-FYVE puncta formation was analyzed. Scale bar, 6 µm. Data represent the mean ± SD from three independent experiments. *P*-value is from the two-sided *t*-test. **g** Human ULK1 structure (PDB code: 4WNP) shows an activation loop domain (yellow). The surrounding areas of R170 and T180 are boxed and enlarged. For the side chain of R170 and T180, nitrogen, oxygen and carbon atoms were shown in purple, red and green, respectively. **h** LN229 cells were incubated under 1% oxygen. **i** Indicated cells were incubated under 1% oxygen for 12 h. **j**, **k** Purified WT Flag-ULK1 (**j**) or/and Flag-ULK1 R170K (**k**) proteins were subjected to a His-PRMT5 protein-mediated in vitro methylation assay and an in vitro autophosphorylation assay. Source data are provided as a Source data file.

We therefore determined whether ULK1 R170me2s affect ULK1 activity under hypoxia. As expected, hypoxia treatment induced ULK1 activity in LN229, Huh7 and HOK cells, while only a limited increase was found in ULK1 R170K mutant (Fig. 3a, Supplementary Fig. 4a). Depletion of endogenous ULK1 and reconstituted expression of ULK1 R170K or a kinase dead ULK1 K46I mutant^35^, but not WT ULK1, substantially abolished ULK1-dependent phosphorylation of Atg13 S355^36^ and Beclin 1 S15^37^ in hypoxia-stimulated LN229, Huh7 and HOK cells (Fig. 3b, Supplementary Fig. 4b). Phosphorylation of GST-Atg13 or His-Beclin 1 by purified WT ULK1 protein, rather than ULK1 R170K mutant protein, was largely enhanced by PRMT5-mediated ULK1 R170me2s (Fig. 3c). Consistently, incubation with WT KDM5C protein inhibited R170me2s-bearing ULK1 protein, which was purified from hypoxia-stimulated LN229 cells, to phosphorylate GST-Atg13 or His-Beclin 1; while this effect was abolished by the catalytic-dead KDM5C H514A mutation (Fig. 3d). Further, knockin expression of ULK1 R170K in LN229 cells or reconstituted expression this mutant in Huh7 or HOK cells eliminated hypoxia-induced activity vacuolar protein sorting 34 (VPS34) in Atg14L immunoprecipitates (Fig. 3e, Supplementary Fig. 4c) without affecting the expression of Beclin 1 and VPS34, and abrogated the formation of EGFP-FYVE puncta (Fig. 3f). These data suggested that ULK1 R170me2s is required for ULK1 activation under hypoxia condition.

A structure analysis (PDB code: 4WNP) revealed that ULK1 R170 is located within a conserved loop domain (Supplementary Fig. 4d), and is spatially closed to T180 (Fig. 3g). Autophosphorylation at ULK1 T180 under stress conditions, such as rapamycin treatment, is required for ULK1 activation^38^. Indeed, immunoblot with a validated specific antibody (Supplementary Fig. 4e) showed a much higher level of T180 phosphorylation under hypoxia treatment (Fig. 3h). Interestingly, glucose or amino acids deprivation also induced similar phosphorylation of ULK1 T180 (Supplementary Fig. 4f). Depletion of endogenous ULK1 and reconstituted expression of a non-phosphoryable ULK1 T180A mutant alleviated hypoxia-stimulated phosphorylation of Atg13 and Beclin 1, activation of VPS34, accumulation of LC3 II, clearance of p62 and formation of LC3–GFP puncta, though no obvious differences was found under normoxia condition (Supplementary Fig. 4g–i).

We, therefore, determined whether ULK1 R170me2s is required for T180 autophosphorylation. Indeed, hypoxia-induced ULK1 T180 phosphorylation was substantially impaired by knockin expression of ULK1 R170K mutation (Fig. 3i). An in vitro kinase assay showed that PRMT5-mediated ULK1 R170me2s was companied with a markedly increased T180 phosphorylation when ULK1 protein was incubated with ATP (Fig. 3j), and this effect was eliminated by ULK1 R170K mutation (Fig. 3k). Consistently, knockdown of PRMT5 in LN229, Huh7, and HOK cells largely abolished hypoxia-induced ULK1 T180 phosphorylation, phosphorylation of Atg13 and Beclin 1, LC3 II accumulation, p62 degradation, and FYVE and LC3 puncta formation (Supplementary Fig. 5a–c). In contrast, transfection of pcDNA3-KDM5C construct, which increased cellular KDM5C expression by 2–4 folds, only limitedly affected these hypoxia-induced effects (Supplementary Fig. 5d–f). Together, these data suggested that hypoxia-repressed KDM5C causes ULK1 R170me2s, and subsequent T180 autophosphorylation-dependent ULK1 activation, which is required for hypoxia-induced autophagy induction.

### ULK1 R170me2s promotes mitochondria turnover and promotes cell growth

**Fig. 4:**
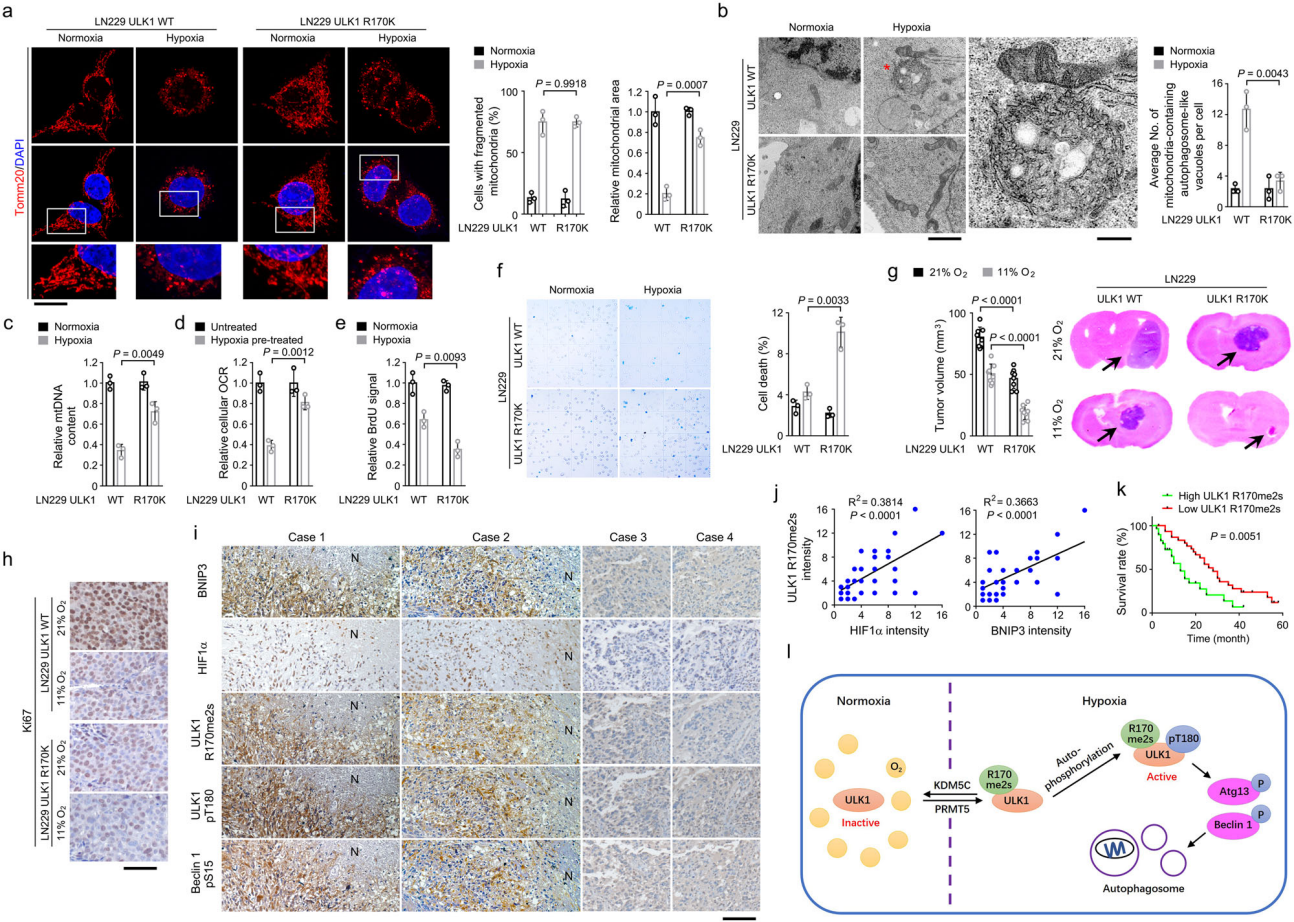
ULK1 R170me2s promotes mitochondria turnover and tumor growth under hypoxia. **c**–**e** Data was normalized to the WT untreated group. **a**, **b** Indicated cells were incubated under 1% oxygen for 12 h. Immunofluorescent staining was performed. The boxed areas (Scale bar, 10 µm) were enlarged (Scale bar, 4 µm) (**a**). Ultrastructure was analyzed. Vacuoles (Scale bar, 1.5 µm) labeled with asterisk were enlarged (Scale bar, 500 nm) (**b**). Data represent the mean ± SD from three independent experiments. *P*-value is from the two-sided *t*-test. **c** Indicated cells were incubated under 1% oxygen for 24 h. The level of mitochondrial DNA (mtDNA) was measured. Data represent the mean ± SD from three independent experiments. *P*-value is from the two-sided *t*-test. **d** Indicated cells were stimulated with 1% oxygen for 18 h, and the cellular OCR was measured immediately in normoxia condition. Data represent the mean ± SD from three independent experiments. *P*-value is from the two-sided *t*-test. **e**, **f** Indicated cells were incubated under 1% oxygen for 48 h. BrdU incorporation assay (**e**) and Trypan blue exclusion assay (**f**) were performed. Data represent the mean ± SD from three independent experiments. *P*-values are from the two-sided *t*-tests. **g** Mice (*n* = 8) were intracranially injected with indicated LN229 cells, and were maintained in ambient atmosphere or 11% oxygen tension before sacrificed for examination of tumor growth (left panel). Representative H&E-stained coronal brain sections are shown (right panel). Data represent the mean ± SD. *P*-values are from the two-sided t-tests. Bonferroni correction was used for multiple hypothesis correction. **h** Immunochemical staining of mice tumors. Scale bar, 75 µm. **i** Immunochemical staining of human GBM tissues. Scale bar, 100 µm. N, necrotic region. **j** Quantification of the immunochemical staining of human GBM samples (*n* = 40). **k** Kaplan–Meier plots of the overall survival time of patients (*n* = 60) with distinct ULK1 R170me2s levels. *P*-value is from the two-sided log-rank test. **l** Model of dynamic ULK1 methylation and autophagy induction under hypoxia. Source data are provided as a Source data file.

Hypoxia induces mitochondria fission, thereby facilitating mitochondrial turnover by autophagy to limit cellular oxygen consumption^39,40^. Immunofluorescent analyses of Tomm20, a mitochondrial marker^41^, revealed that hypoxia treatment induced comparable mitochondrial fragmentation in LN229 cells with either WT ULK1 or ULK1 R170K mutant (Fig. 4a). Notably, ULK1 R170K mutation largely restored the Tomm20-positive mitochondrial area in hypoxia-stimulated cells (Fig. 4a), hinting that ULK1 R170me2s probably facilitates the degradation of mitochondria. Indeed, hypoxia treatment markedly enhanced the engulfment of mitochondria in autophagosome-like vacuoles in WT LN229 cells shown by TEM-based ultrastructure analyses, which was substantially abolished by ULK1 R170K mutation (Fig. 4b). Further, ULK1 R170K mutation or knockdown of PRMT5 partially rescued the loss of mitochondrial DNA (mtDNA) in hypoxia-stimulated LN229, Huh7, and HOK cells, suggesting an impaired mitochondrial turnover (Fig. 4c, Supplementary Fig. 6a, b). Given that mitochondria are the major oxygen-consuming organelles for mammalian cells^42^, we incubated cells with 1% oxygen for 18 h, and measured the cellular oxygen consumption rate (OCR) immediately in normoxia condition. As expected, hypoxia treatment resulted in about 70% decrease in cellular OCR in LN229, Huh7 and HOK cells (Fig. 4d, Supplementary Fig. 6c, d). In contrast, either ULK1 R170K mutation or PRMT5 depletion restored cellular OCR, though it did not apparently alter cellular OCR without hypoxia stimulation (Fig. 4d, Supplementary Fig. 6c, d). However, the level of mtDNA or cellular OCR was not obviously changed upon overexpression of KDM5C (Supplementary Fig. 6e, f). In line with this, BrdU incorporation assay showed that mutation of ULK1 R170K or reconstituted expression of Atg13 S355A/Beclin 1 S15A further reduced hypoxia-repressed proliferation (Fig. 4e, Supplementary Fig. 6g, h). In addition, an increased cell death was also observed in ULK1-mutated LN229 cells upon hypoxia treatment by trypan blue exclusion assay (Fig. 4f). These results suggested that ULK1 R170me2s plays a crucial role in mitochondrial turnover and maintains cell viability in response to hypoxia stress.

To find out the impact of ULK1 R170me2s on tumor development, we intracranially injected athymic nude mice with WT LN229 cells or LN229 cells with knockin expression ULK1 R170K. Compared to WT LN229 cells, ULK1 R170K mutation inhibited tumor growth (Fig. 4g) with decreased cell proliferation (Fig. 4h) and elevated cell death (Supplementary Fig. 6i). Immunohistochemical staining with validated anti-ULK1 R170me2s or anti-ULK1 pT180 antibody (Supplementary Fig. 6j, k) revealed that ULK1 R170K mutation abolished ULK1 R170me2s, and reduced the phosphorylation of ULK1 T180, Atg13 S355, and Beclin 1 S15 in the tumor samples derived from the mice in normoxia condition (Supplementary Fig. 6l). Notably, exposure of the tumor-bearing mice to 11% low oxygen environment substantially impaired tumor growth (Fig. 4g). Such low oxygen availability resulted in elevated expression of hypoxia markers HIF1α and BNIP3^43,44^, increased ULK1 R170me2s, as well as enhanced phosphorylation of ULK1 T180, Atg13 S355, and Beclin 1 S15 (Supplementary Fig. 6l). Notably, expression of ULK1 R170K mutation attenuated low-oxygen-induced phosphorylation of ULK1 T180, Atg13 S355 and Beclin 1 S15, and accordingly further curbed tumor growth (Fig. 4g) with repressed cell proliferation (Fig. 4h) and increased cell death (Supplementary Fig. 6i). To further expand our findings, we injected fluorescent probe-labeled HOK cells into the pericardial cavity of zebrafish embryos (Supplementary Fig. 6m)^45^. As expected, ULK1 R170K mutation further reduced the number of cells in zebrafish that were maintained under hypoxia condition (Supplementary Fig. 6n). These results suggested that ULK1 R170me2s copes with hypoxia stress to maintain cell viability.

To evaluate the clinical relevance of ULK1 R170me2s, human glioblastoma samples was analyzed by immunohistochemical staining. We show that the level of HIF1α, BNIP3, ULK1 R170me2s, ULK1 pT180, and Beclin 1 pS15 were positively corelated (Fig. 4i, Supplementary Fig. 6o). Quantification of staining intensities revealed that these correlations were significant (Fig. 4j, Supplementary Fig. 6p). Notably, a significantly shorter survival time was found in those GBM patients with a high level of R170me2s (Fig. 4k). These results underscore the clinical relevance of ULK1 R170 me2s in tumor development.

## Discussion

Oxygen concentration is important for tissue homeostasis. As a result of low oxygen availability, the growth and viability of cells is reduced^46^. Autophagy is one of the activated cellular responses under multiple stressful conditions, including hypoxia^5^. Nevertheless, how the early steps during autophagy induction are regulated in a hypoxic environment was relatively unknown. In present study, we show that oxygen-sensitive dimethylation of ULK1 R170 is accumulated under hypoxia, which facilitates activation of ULK1, phosphorylation of ULK1 substrates Atg13 and Beclin 1, and subsequent formation of autophagosomes. Autophagy-dependent clearance of mitochondria reduced oxygen consumption, render cells resistant to low oxygen tension, and maintain cell viability (Fig. 4l).

Arginine methylation is a prevalent post-translational modification found on both nuclear and cytoplasmic proteins. This modification enables covalent addition of methyl groups to the side chain of arginine residues in a protein, predominantly in three types in eukaryotic cells: mono, symmetric and asymmetric. Methylation and demethylation of arginine residues is a dynamic cyclical process, and a well-balanced arginine methylation is required for cell proliferation and survival^47–49^. In the present data, we demonstrate that ULK1 R170me2s status was dynamically controlled by a methylation/demethylation system, which consist of PRMT5 and KDM5C. PRMT5, formerly termed Janus kinase-binding protein 1, that catalyzes the formation of mono-methylarginine and symmetric dimethylarginine in a variety of proteins^50^. In contrast, KDM5C is a member of 2-oxoglutarate-dependent JmjC methylysine demethylases, which has been recently evidenced as an arginine demethylase^32^. Our data show that ULK1 could be methylated by PRMT5 at R170, which is insusceptible to oxygen concentration. Notably, ULK1 R170me2s was largely accumulated under hypoxia condition due to the repressed-KDM5C, which use oxygen as a cofactor to catalyze arginine demethylation through an oxidation reaction.

It is widely accepted that both mTOR and AMPK regulate ULK1 activity in different ways. When sufficient nutrients exist, mTOR stays active, which suppressed autophagy initiation by phosphorylating and blunting ULK1^51^. In response to glucose deprivation, AMPK is activated in case of low ATP levels, and in turn activates ULK1 by releasing it from mTOR^22^. Though repressed mTOR and active AMPK was previously detected under hypoxia condition, our results show that accumulation of LC3 II and formation of LC3–GFP puncta occurs in prior to mTOR or AMPK-mediated ULK1 phosphorylation, which suggested that mTOR or AMPK was not likely the major regulator for hypoxia-induced autophagy.

HIF1α plays a crucial role in the regulation of oxygen homeostasis. HIF1α is hydroxylated at proline 402/564 under normoxia condition, which could be recognized by von Hippel Lindau protein, leading to the recruitment of ubiquitin protein ligase complexes that target HIF1α for proteasomal degradation. Under hypoxic conditions, HIF1α hydroxylation is largely abolished, since oxygen is utilized as a cofactor for this reaction. Stabilized HIF1α transactivates a variety of hypoxia-responsive genes, therefore contributing to the cell adaptation to hypoxia^52^. Particularly, HIF1α was found to be essential for hypoxia-mediated autophagy through promoting the expression of BNIP3 and BNIP3L, two BH3 domain-containing proteins, to release Beclin 1 from Bcl2^53^. In the present data, however, HIF1α shows minor effects on hypoxia-induced ULK1 R170me2s, suggesting that PRMT5 and KDM5C governs ULK1 R170me2s in an HIF1α-independent manner. Collectively, it is likely that ULK1 methylation and HIF1α hydroxylation synergistically regulates autophagy induction in response to hypoxic stress, by promoting the activation of ULK1 and Beclin 1-containing VPS34 complex and, respectively.

Autophagy, which enables the recycling of nutrients, amino acids and lipids, are often hijacked by tumor cells to counteract with stressful environment^54^. In this study, our findings illustrate a novel and important mechanism responsible for hypoxia-induced autophagy. Hypoxia-repressed KDM5C accumulates PRMT5-mediated ULK1 R170me2s, which results in ULK1 activation and autophagy induction. Inhibition of ULK1 R170me2s abolished hypoxia-triggered autophagy and impaired brain tumor development.

## Methods

### Materials

Rabbit polyclonal antibody recognizing phosphorylated ULK1 pT180 (1:1000), ULK1 R170me2s (1:1000) and ULK1 R170me2a (1: 1000) were customized from Boer Biotechnology (Chengdu, China). Peptides containing ULK1 pT180, ULK1 R170me2s, or ULK1 R170me2a were injected into rabbits. The rabbit serum was collected and purified using an affinity column conjugated with non-modified peptide to exclude antibodies recognizing non-modified ULK1, followed by an affinity column conjugated with ULK1 pT180, ULK1 R170me2s or ULK1 R170me2a peptide to bind to and purify the antibodies. Antibodies were then eluted and concentrated.

Antibodies recognizing ULK1 (#8054, 1:1000), HIF1α (#36169, 1:1000), Tubulin (#2125, 1:2000), LC3B (#83506, 1:1000), p62 (#88588, 1:1000), Beclin 1 (#4122, 1:1000), Beclin 1 pS15 (#84966, 1:500), Atg13 (#13468, 1:1000), Atg13 pS355 (#46329, 1:500), S6K pT421/pS424 (#9204, 1:1000), Tomm20 (#42406, 1:500), TSC1 (#6935: 1:500) and AMPKα (#5832, 1:1000) were obtained from Cell Signaling Technology. Antibodies recognizing ULK2 (ab97695, 1:1000), PRMT5 (ab109451, 1:1000), KDM5C (ab194288, 1:1000), BNIP3 (ab109362, 1:1000), S6K (ab32529, 1:1000), ACC (ab109368, 1:1000), ACC pS79 (ab68191, 1:500), ULK1 pS757 (ab229909, 1:500), and ULK1 pS555 (ab229537, 1:500) were obtained from Abcam. Antibody recognizing KDM4E (ABE1081, 1:1000) was obtained from Merck. Antibody against Flag (F2555, 1:5000), anti-Flag M2 agarose beads, streptavidin-conjugated agarose beads, a-ketoglutarate, dimethyl-αKG, DAPI, ATP, chloroquine and bovine serum albumin were purchased from Sigma. GFP-Trap beads were purchased from ChromoTek. IOX1 was purchased from Selleck Chemicals. HyFect transfection reagents were obtained from Denville Scientific. Recombinant Atg13 and Beclin 1 fusion proteins were purchased from Proteintech Group. [γ-^32^P]-ATP was purchased from MP Biomedicals.

### DNA constructs and mutagenesis

PCR-amplified human ULK1, ULK2, KDM5C, PRMT5, Atg13, Beclin 1 and Atg14L were subcloned into pcDNA3.1/hygro(+)-Flag, pcDNA3.1/hygro(+)-HA, pCDH-SFB, or pCold I (His) vector, respectively. Mutants were constructed using the QuikChange site-directed mutagenesis kit (Stratagene, La Jolla, CA). shRNA-resistant Beclin 1 contains mutation of c1332a, a1335c, a1338g and t1341c.

The following pGIPZ shRNAs were used: control shRNA, GCT TCT AAC ACC GGA GGT CTT; human ULK1 shRNA, TGC AAA GAA AAA CAC ACG T (targeting non-coding region); mouse ULK1 shRNA, AGT CTG CGT ACC ACC TGC T (not recognizing human ULK1); KDM5C shRNA #1, TTC ATA GGG ATA AAC AAT G; KDM5C shRNA #2, TTA TTA CAA CAT GAA CCC A; PRMT5 shRNA, AGT GTG TCA GCT ATT TCG G; HIF1α shRNA, TAT AAA TAG ACT GCT TTA G; Beclin 1 shRNA, AAA ATT GTG AGG ACA CCC A. Atg13 shRNA was purchased from Sigma (Clone ID:NM_014741.3-2259s21c1, targeting non-coding region).

### Cell culture and hypoxia treatment

LN229 GBM cells and 293T cells were obtained from ATCC. Huh7 HCC cells were obtained from JCRB Cell Bank. HOK cells were obtained from ScienCell. LN229, Huh7, and indicated MEF cells were maintained in Dulbecco’s modified Eagle’s medium supplemented with 10% fetal bovine serum. HOK cells were maintained in Oral Keratinocyte Medium (OKM, Cat. No. 2611). For generating shRNA-depleted stable cell lines, cells were transfected with shRNA plasmids and selected by puromycin. For generating gene-expressing stable cell lines, shRNA-depleted cells were infected with lentivirus carrying WT ULK1, ULK1 R170K or ULK1 T180A and selected by hygromycin. For hypoxia treatment, cells were incubated in a Ruskinn InvivO2 workstation under 1% oxygen concentration for indicated time, unless other oxygen concentrations were indicated.

### Immunofluorescent staining

Cells were fixed and incubated with primary antibodies at a dilution of 1:100, fluorescence dye-conjugated secondary antibodies, and DAPI, according to standard protocols^55^. Cells were examined using a laser confocal microscope (Zeiss, Thornwood, NY). Cells were classified as having fragmented mitochondria when more than 50% of the total cellular organelles displayed a major axis shorter than 3 µm^56^. The mitochondrial areas were quantified using Fiji software by calculating the Tomm20-positive areas of 200 cells.

### Mass spectrometric analysis

Protein bands visualized via Coomassie Brilliant Blue staining was excised from SDS-PAGE gel and digested in 50 mM ammonium bicarbonate buffer containing RapiGest (Waters Corporation) overnight at 37 °C with 200 ng of modified sequencing-grade trypsin (Promega). The digested samples were analyzed using high-sensitivity liquid chromatography tandem mass spectrometry with an Orbitrap Fusion Lumos mass spectrometer (Thermo Fisher Scientific).

### BrdU incorporation assay

BrdU incorporation assay was performed using Cell Proliferation ELISA, BrdU (colorimetric) (Roche) following the manufacture’s instruction. Briefly, cells were cultured in a 96-well microplate with 100 μl/well medium. 10 μl/well BrdU labeling solution was added in the culture and the cells were incubated for 8 h at 37 °C. After the labeling medium was removed, the cells were incubated with 200 μl/well FixDenat at 25 °C for 30 min, 100 μl/well Anti-BrdU-POD working solution at 25 °C for 90 min, and then 100 μl/well Substrate Solution at 25 °C for 20 min. The absorbance of the samples was measured at 370 nm.

### Trypan blue exclusion assay

0.1 ml of the 0.4% trypan blue stock solution (Thermo Fisher Scientific) was added to 0.9 ml cell suspension. 3 min later, 10 µl cell suspension was loaded onto a hemacytometer, and the cells were examined immediately under a microscope at low magnification.

### TUNEL assay

TUNEL assay was performed using TUNEL Assay Kit-HRP-DAB (Abcam) following the manufacture’s instruction. Briefly, the specimen was sequentially incubated with Proteinase K at room temperature for 20 min, 3% hydrogen peroxide at room temperature for 5 min, TdT Equilibration Buffer at room temperature for 30 min, TdT Labeling Reaction Mix at 30 °C for 90 min, Stop Buffer at room temperature for 5 min, Blocking Buffer at room temperature for 10 min, and Conjugate solution at room temperature for 30 min. The TUNEL signal was developed by incubation with DAB working solution at room temperature for 15 min.

### Genomic editing

Genomic mutations in LN229 cells were created using the CRISPR/Cas9 system^57^. In a 24-well plate, LN229 cells at 60% confluence were co-transfected with a donor template to introduce the mutations and a vector to express sgRNA and WT hSpCas9 tagged with green fluorescent protein. 48 hours after transfection, cells were trypsinized and diluted in a medium for single-cell seeding into a 96-well plate, and green fluorescent protein-positive cells were marked and subjected to genomic DNA extraction.

### Measurement of mitochondrial DNA content

Total DNA was extracted by using the QIAamp DNA mini kit (QIAGENE, Germantown, MD) according to the manufacturer’s instructions. The levels of mitochondrial DNA D-Loop structure were measured by qRT-PCR, and normalized to genomic DNA-encoded β-actin. The sequences of the primers used were listed in Supplementary Table 1.

### Immunoprecipitation and immunoblot analysis

Extraction of proteins from cultured cells was performed with a lysis buffer (50 mM Tris-HCl [pH 7.5], 0.1% SDS, 1% Triton X-100, 150 mM NaCl, 1 mM dithiothreitol, 0.5 mM EDTA, 100 μM PMSF, 100 μM leupeptin, 1 μM aprotinin, 100 μM sodium orthovanadate, 100 μM sodium pyrophosphate, and 1 mM sodium fluoride). Cell extracts were clarified by centrifugation at 13,400 × *g*, and the supernatants (2 mg protein/ml) were subjected to immunoprecipitation with the indicated antibodies. After overnight incubation at 4 °C, protein A or G agarose beads were added and left for an additional 3 h. Immunocomplexes were washed with lysis buffer 3 times and then subjected to immunoblot analyses with corresponding antibodies^58^. The band intensity was quantified using the Image Lab software program (Bio-Rad).

### Purification of recombinant proteins

Expression of PRMT5 proteins were induced in bacteria^59^. Briefly, BL21(DE3) cells expressing PRMT5 proteins were cultured in 250 ml of LB medium and treated with isopropyl β-D-1-thiogalactopyranoside (IPTG) for 16 h at 30 °C before lysis via sonication.

ULK1 and KDM5C proteins were expressed in 293 T cells. Briefly, a pcDNA3.1 plasmid (10 μg) expressing recombinant proteins with tags were transfected into 293 T cells seeded in a 15 cm-diameter dish with confluence of 70%. 48 h after transfection, the cells were incubated with fresh DMEM supplemented with 10% bovine calf serum for 1 h and then harvested with a non-denaturing lysis buffer (20 mM Tris-HCl pH 8.0, 137 mM NaCl, 1% NP-40).

For purification of His-tagged proteins, cell lysates were loaded onto a Ni-NTA column (GE Healthcare Life Sciences) followed by washing with five column volumes of 20 mM imidazole and subsequent elution with 250 mM imidazole.

For purification of Flag-tagged proteins, cell lysates were loaded onto a column containing Anti-Flag M2 agarose affinity gel followed by washing with five column volumes of phosphate-buffered saline (PBS) and subsequent elution with 100 μg/ml Flag peptide. The proteins in 10-kDa spin columns were desalted via washing twice with ice-cold PBS.

The proteins were then loaded onto a HiPrep 16/60 Sephacryl S-200 HR gel filtration column (GE Healthcare Life Sciences) to remove contaminated proteins. The purification efficiency was examined using SDS-PAGE and colloidal Coomassie Brilliant Blue (G-250) staining, with superior sensitivity (as low as 1 ng of protein per band)^59^.

### TEM

Cells were fixed in 0.1% glutaraldehyde in 0.1 M sodium cacodylate for 2 h, postfixed with 1% OsO4 for 1.5 h, washed and finally stained for 1 h in 3% aqueous uranyl acetate. The samples were then rinsed with water again, dehydrated with graded alcohol (50%, 75% and 95–100% alcohol) and embedded in Epon-Araldite resin (Canemco, 034). Ultrathin sections were cut on a Reichert Ultramicrotome, counterstained with 0.3% lead citrate and examined on a Philips EM420 transmission electron microscope. The cells with autophagic vacuoles were defined as cells that had 5 or more autophagic vacuoles^60^. Values for the area occupied by autophagic vacuoles and the cytoplasm were obtained with Image Pro Plus version 3.

### In vitro methylation assay

In vitro methylation assay was performed according to the previous report^61^. Briefly, 500 ng purified PRMT5 protein and 200 ng purified ULK1 protein were mixed with 50 μl methylation buffer (20 mM Tris [pH 8.8], 4 mM EDTA, 1 mM PMSF, 0.5 mM DTT and 5 mM SAM), and incubated at 30 °C for 1 h. The reaction was stopped by adding SDS loading buffer, and the proteins were resolved on SDS-PAGE gels.

### In vitro demethylation assay

500 ng purified KDM5C protein and 200 ng purified ULK1 protein were incubated in 100 μl of demethylase reaction buffer containing 20 mM Tris-HCl (pH 7.5), 150 mM KCl, 50 mM Fe(NH_4_)_2_(SO_4_)_2_, 1 mM a-ketoglutarate, and 1 mM ascorbate for 1 h at 37 °C^62^. Reactions were terminated by boiling for 5 min in SDS sample loading buffer.

### ULK1 kinase assay

In vitro ULK1 kinase assay was performed following previous report^15^. For ULK1-mediated phosphorylation of Atg13 or Beclin 1, 20 ng modified or non-modified ULK1 proteins were mixed with 10 ng Atg13 or 10 ng Beclin 1 proteins were incubated in 25 µl of kinase buffer (50 mM Tris-HCl [pH 7.5], 100 mM KCl, 50 mM MgCl_2_, 50 mM MnCl_2_, 1 mM Na_3_VO_4_, 1 mM DTT, 5% glycerol, 0.5 mM ATP) at 37 °C for 1 h. For ULK1 autophosphorylation, 5 ng modified or non-modified ULK1 proteins were incubated in 25 µl of kinase buffer at 37 °C for 10 min. ULK1 activity assay was performed using ADP-Glo™ Kinase Assay + ULK1 Kinase Enzyme System (Promega, Madison, WI, USA)^63^.

### VPS34 lipid kinase assay

Atg14L-associated VPS34 complex was immunoprecipitated, washed with PBS, followed by three washes with the reaction buffer. The protein complex was incubated in 60 µL of reaction buffer (10 mM Tris-HCl [pH 7.4], 100 mM NaCl, 1 mM EDTA) supplemented with 10 mM MnCl_2_, 20 µg phosphatidylinositol, 0.5 mM ATP, and 10 µCi [γ-^32^P]ATP at ambient temperature for 30 min. The reaction was quenched by adding 20 µL of 8 M HCl and extracted with 160 µL chloroform/methanol (1:1). Extracted phospholipid products were separated on TLC using a coated silica gel and a solvent composed of chloroform/methanol/H_2_O/NH_4_OH (v/v/v/v, 9:7:1.7:0.3). The TLC plates were dried and exposed by autoradiography to visualize PI(3)P production.

### Measurement of cellular OCR

Briefly, 3 × 10^4^ cells were plated onto XF24 plates in DMEM (0.5% BCS, 10 mM glucose, 2 mM glutamine) (Seahorse Bioscience, North Billerica, MA) and incubated at in 21% or 1 % oxygen tension for indicated time. The medium was then replaced with 675 μl of unbuffered assay medium (Seahorse Bioscience) supplemented with 2 mM glutamine, 10 mM glucose (pH was adjusted to 7.4 using 0.5 mM sodium hydroxide). Cellular OCR were recorded using the XF24 plate reader. Homogeneous plating and cell counts were assessed by fixing the cells with 10% trichloroacetic acid for 1 h at 4 °C and then staining the fixed cells with a 0.47% solution of sulforhodamine B^64^.

### Mouse xenograft model

Four-week-old female athymic nude BALB/c mice were used in this study. Mice were housed under ambient temperature of 24 ± 2 °C, circulating air, constant humidity of 50 ± 10% and a 12 h:12 h light/dark cycle. 1 × 10^5^ cells with knockin mutations were suspended in 5 μl of Dulbecco’s modified Eagle’s medium into mice (*n* = 8). For hypoxia treatment, mice were raised in a controlled oxygen tension (11%) chamber. Animals were sacrificed 28 days after injection. The brain of each mouse was dissected, which is followed by fixation in 4% formaldehyde and embedment in paraffin. The tumor volume was calculated using the formula: *V* = ½*a*^2^*b* (*V*: volume, *a*: shortest diameter, *b*: longest diameter). The animals were treated in accordance with relevant institutional and national guidelines and regulations. The use of animals was approved by the institutional review board of Chengdu Medical College.

### Zebrafish xenograft model

Zebrafish xenograft was established using embryos of Tg(flk1: EGFP) zebrafish strain obtained 48 hours postfertilization, following previous report^65^. Zebrafish were maintained under temperature at 28 °C, pH 7.2–7.4, and a 14 h-on/10 h-off light cycle. Cells were labeled with 2 µM CellTracker™ CM-Dil Dye (Thermo Fisher Scientific), washed with PBS, and loaded into a borosilicate glass needle pulled by a Flaming/Brown micropipette puller (Narishige, Japan, PN-30). Zebrafish embryos, 48 h postfertilization, were anesthetized by 0.003% tricaine. 20 nL suspension containing about 15 cells were implanted into the pericardial cavity of zebrafish embryo by using an electronically regulated air-pressure microinjector (Harvard Apparatus, NY, PL1-90). After injection, digital micrographs were taken with a Zeiss Imager. Z1 fluorescence microscope (Carl Zeiss Microimaging Inc., Germany) equipped with an AxioCam MRc5 digital CCD camera (Carl Zeiss Microimaging Inc., Germany). The number of CM-Dil-labeled cells was evaluated by automated cell counting via custom^66,67^. Those zebrafish embryos with 15 cells were selected for each group. Zebrafish were maintained in a 1-liter standard plastic aquarium with a lid. For hypoxia treatment, nitrogen gas tank was switched on and the air saturation level shown on the oxygen regulator was set to 7.5%^45^. 5 days after infection, zebrafish without cell dissemination to the posterior part were selected (*n* = 10 for each group), and the CM-Dil-labeled cells were quantified.

### Histologic evaluation and immunohistochemical staining

The immunohistochemical staining was performed using the VECTASTAIN ABC kit (VECTOR LABORATORIES, CA) according to manufacturer’s instructions. We quantitatively scored the tissue sections according to the percentage of positive cells and staining intensity, as previously defined^68^. We assigned the following proportion scores: 0 if 0% of the tumor cells showed positive staining, 1 if 0% to 10%, 2 if 11% to 30%, 3 if 31% to 70%, and 4 if 71% to 100%. We also rated the intensity of staining on a scale of 0 to 4: 0, negative; 1, weak; 2, moderate; 3, strong; 4, very strong. We then multiply the proportion and intensity scores to obtain a total score (range: 0-16). The use of human GBM tumor specimens was approved by the Institutional Review Board at Chengdu Medical college.

### Statistics and reproducibility

All experiments were repeated independently with similar results for three times, and all data represent the mean ± standard deviation (SD) of three independent experiments, unless otherwise specified. Differences in means were considered statistically significant at *P* < 0.05. Bonferroni correction was used for multiple hypothesis correction (requiring *p* < 0.05/N, with N indicating the number of comparisons) to prevent the reporting of false positive results. GraphPad Prism 7 software was used for statistical analyses.

### Reporting summary

Further information on research design is available in the Nature Research Reporting Summary linked to this article.

## Supplementary information

Supplementary Information

Reporting Summary

## Data Availability

All the data supporting the findings of this study are available within the article and its Supplementary information files. The proteomic data generated in this study have been deposited in the ProteomeXchange Consortium via the iProX proteomic data repository under the accession number PXD031209. All relevant data are available upon reasonable request. Source data are provided with this paper.

## Source data

Source data
