# Supplementary material for: Oxygen-sensitive methylation of ULK1 is required for hypoxia-induced autophagy
Source: Nat Commun. 2022 Mar 4;13:1172. doi: 10.1038/s41467-022-28831-6 (PMC8897422; doi:10.1038/s41467-022-28831-6)
Supplement: Supplementary file 1 — Supplementary Information [file 41467_2022_28831_MOESM1_ESM.pdf]

Supplementary Information

Oxygen-sensitive methylation of ULK1 is required for hypoxia-induced autophagy

Jingyi Li, Tao Zhang, Tao Ren, Xiaoyu Liao, Yilong Hao,

Je Sun Lim, Jong-Ho Lee, Mi Li, Jichun Shao, Rui Liu

Supplementary Figure 1

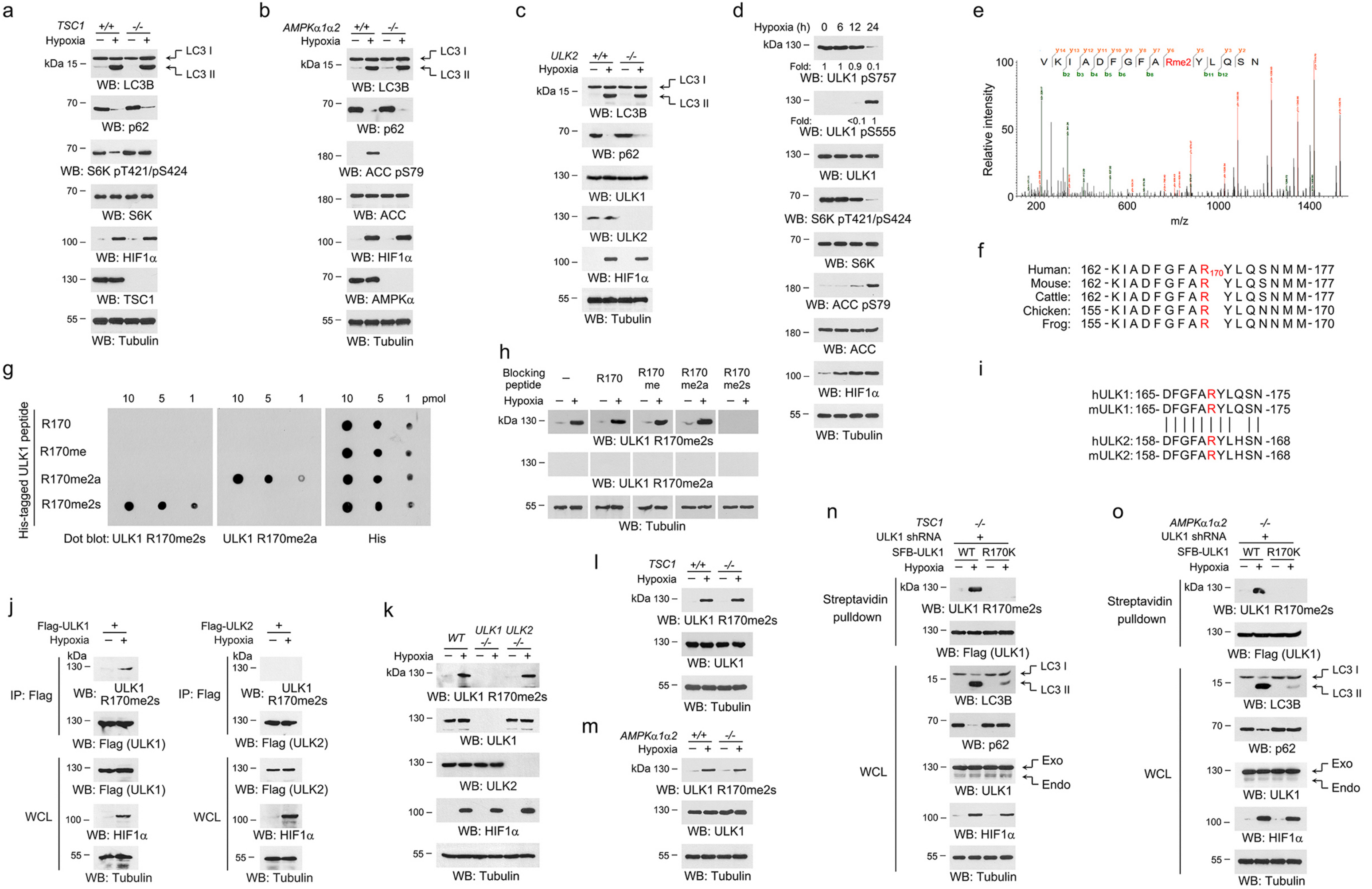

Supplementary Figure 1 Hypoxia induce ULK1 R170me2s

(a-d, g, h, j-o) Immunoblot was performed with the indicated antibodies.

(a-c) WT, *TSC1* -/- (a), *AMPKα1α2* -/- (b), or *ULK2* -/- MEFs (c) were incubated under 1% oxygen for 24 h.

(d) LN229 cells were incubated under 1% oxygen for indicated time.

(e) Flag-ULK1 protein purified from hypoxia-stimulated LN229 cells was subjected to mass spectrometry. Mass spectrometric analysis was performed on a tryptic fragment that matched to the peptide VKIADFGFARYLQSN with dimethylation on arginine.

(f) Alignment analysis of the spanning sequences of ULK1 R170 among species.

(g) Dot blot analyses of the anti-ULK1 R170me2s and anti-ULK1 R170me2a against the His-tagged unmodified or modified ULK1 peptides (aa 162-177).

(h) LN229 cells were incubated under 1% oxygen for 12 h. Immunoblotting was performed in presence or absence of indicated peptides.

(i) Alignment analysis of ULK1 R170 spanning sequences.

(j) LN229 cells with expression of Flag-ULK1 or Flag-ULK2 were incubated under 1% oxygen for 12 h. Immunoprecipitation was performed using an anti-Flag antibody.

(k) WT, *ULK1* -/-, or *ULK2* -/- MEFs were cultured under 1% oxygen for 12 h.

(l-m) WT, *TSC1* -/- (l), *AMPKα1α2* -/- (m) MEFs were cultured under 1% oxygen for 12 h.

(n-o) Endogenous *ULK1*-depleted *TSC1* -/- (n) or *AMPKα1α2* -/- (o) MEFs with reconstituted expression of human WT SFB-ULK1 or SFB-ULK1 R170K were incubated under 1% oxygen for 12 h. A streptavidin pulldown was performed. ShRNA targeting mouse *ULK1* does not recognize human ULK1.

Source data are provided as a Source Data file.

Supplementary Figure 2

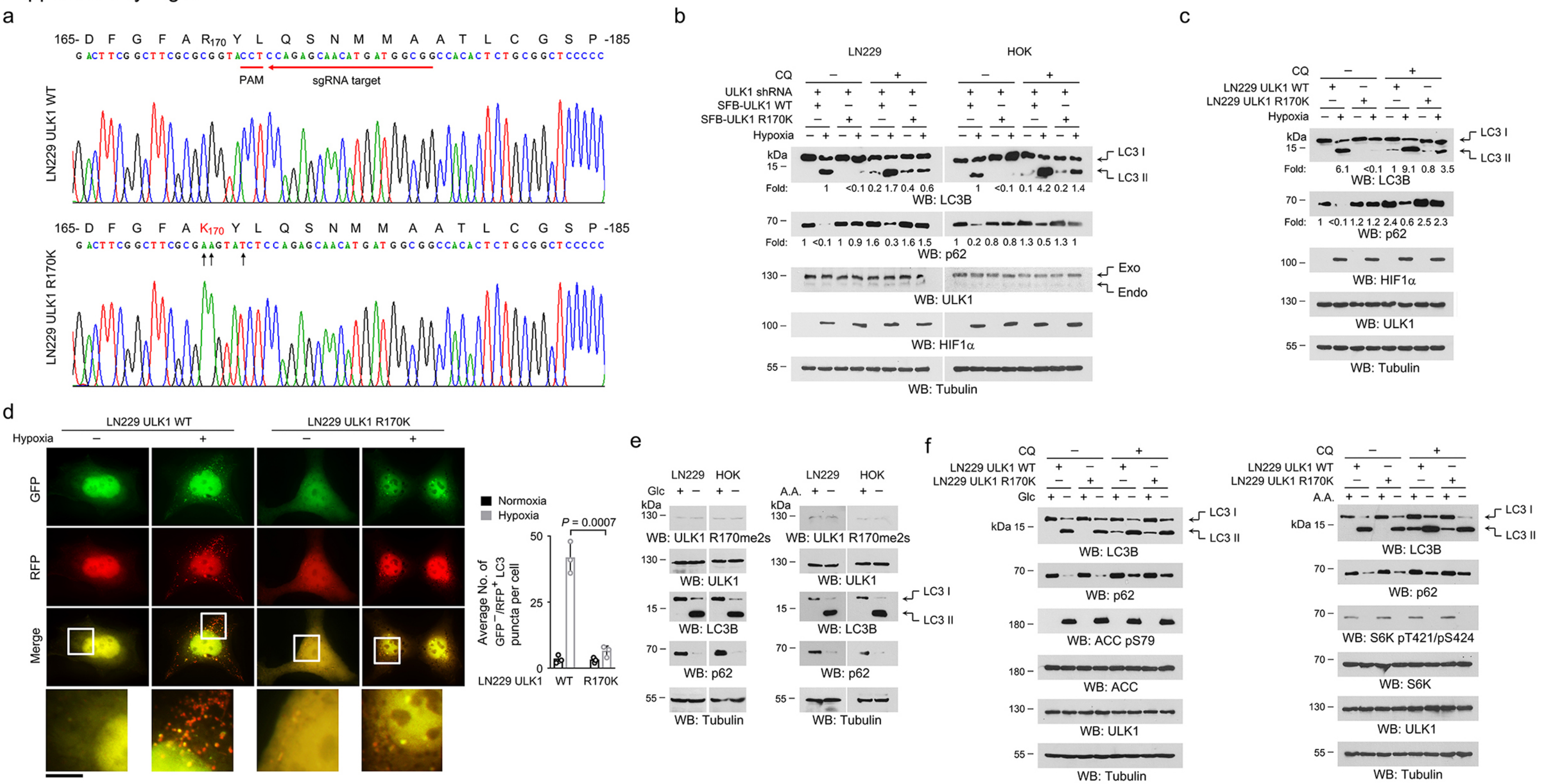

Supplementary Figure 2 ULK1 is R170me2s required for hypoxia-induced autophagy

(a) Sequencing of WT LN229 and individual clone of ULK1 R170K mutant. The red line indicates sgRNA-targeting sequence and the protospacer adjacent motif. The mutated nucleotides are highlighted by arrows. The mutated amino acids are highlighted in red.

(b) Endogenous ULK1-depleted LN229 or HOK cells with reconstituted expression of WT SFB-ULK1 or SFB-ULK1 R170K were incubated under 1% oxygen for 12 h in presence or absence of 20 μM CQ.

(c) WT LN229 cells or LN229 cells with knockin expression of ULK1 R170K mutant were incubated under 1% oxygen for 12 h in presence or absence of 20 μM CQ.

(d) WT LN229 cells or LN229 cells with knockin expression of ULK1 R170K mutant were transfected with LC3-RPF-GFP plasmid. 48 h after transfection, cells were incubated under 1% oxygen for 12 h, and the fluorescent LC3 puncta were analyzed. Data represent the mean ± SD from 3 independent experiments. *P*-value is from the two-sided t-test. Scale bar: 8 μm for original images, 2.5 μm for enlarged images.

(e) LN229 and HOK cells were stimulated with glucose deprivation for 2 h or amino acid deprivation for 4 h.

(f) WT LN229 cells or LN229 cells with knockin expression of ULK1 R170K mutant were stimulated with glucose deprivation for 2 h (left panel) or amino acid deprivation for 4 h (right panel) in presence or absence of 20 μM CQ.

Source data are provided as a Source Data file.

Supplementary Figure 3

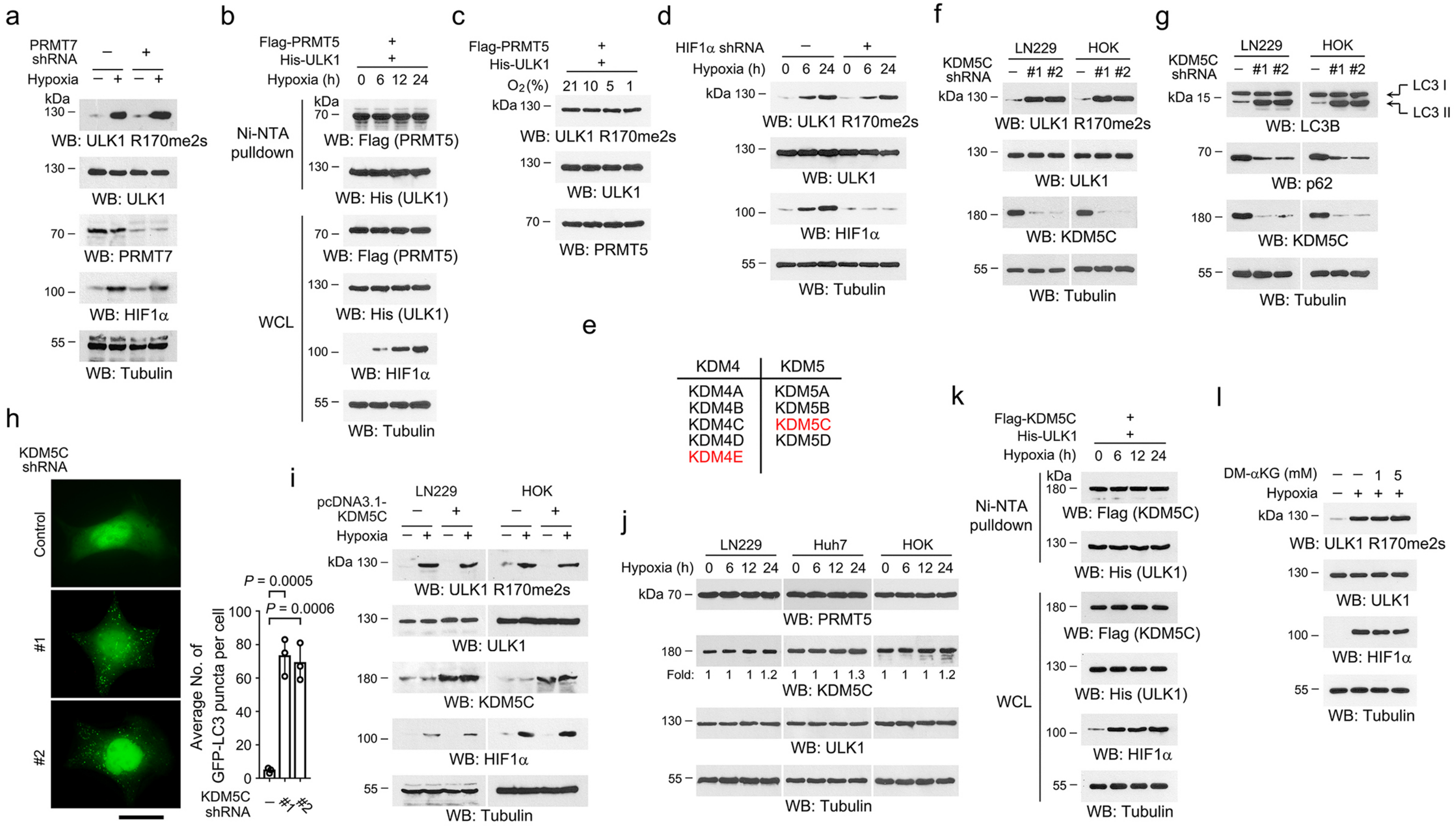

Supplementary Figure 3 ULK1 R170 is symmetrically dimethylated by PRMT5 and demethylated by KDM5C

(a-d, f, g, i-l) Immunoblot was performed with the indicated antibodies.

(a) Endogenous PRMT7-depleted LN229 cells were incubated under 1% oxygen for 12 h.

(b) LN229 cells with expression of Flag-PRMT5 and His-ULK1 were incubated under 1% oxygen for indicated time. A Ni-NTA beads pulldown was performed.

(c) Purified Flag-PRMT5 protein was mixed with purified His-ULK1 protein for an *in vitro* methylation assay in presence of indicated concentrations of oxygen.

(d) Endogenous HIF1α-depleted LN229 cells were incubated with 1% oxygen for indicated time.

(e) Members of KDM4 and KDM5 families. KDM4E and KDM5C (red) were reported to have the arginine demethylation activity on the histone peptides.

(f-g) LN229 and HOK cells with expression of two distinct KDM5C shRNAs were cultured in normoxia condition.

(h) LN229 cells with expression of two distinct KDM5C shRNAs and LC3-GFP were cultured in normoxia condition. Scale bar, 8 μm. Data represent the mean ± SD from 3 independent experiments. *P*-values are from the two-sided t-tests. Bonferroni correction was used for multiple hypothesis correction.

(i) LN229 and HOK cells with expression of pcDNA3.1-KDM5C were incubated under 1% oxygen for 12 h.

(j) LN229, Huh7 and HOK cells were incubated under 1% oxygen for indicated time.

(k) LN229 cells with expression of Flag-KDM5C and His-ULK1 were incubated under 1% oxygen for indicated time. A Ni-NTA pulldown was performed.

(l) LN229 cells treated with indicated concentrations of DM-αKG were incubated under 1% oxygen for 12 h.

Source data are provided as a Source Data file.

Supplementary Figure 4

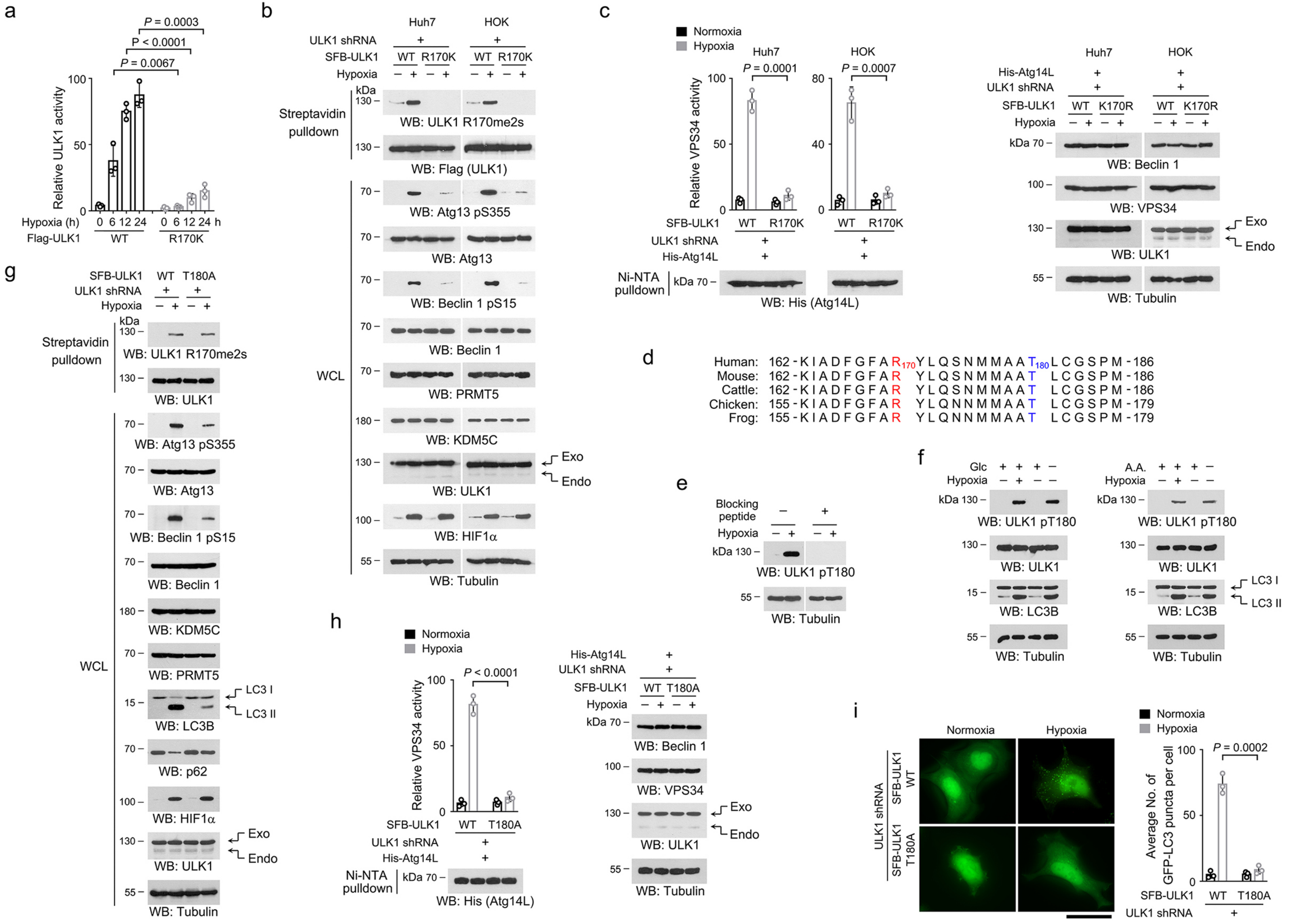

Supplementary Figure 4 R170me2s activates ULK1 by facilitating ULK1 T180 autophosphorylation

(b, c, e-h) Immunoblot was performed with the indicated antibodies.

(a) Huh7 cells with expression of WT Flag-ULK1 and Flag-ULK1 R170K were incubated under 1% oxygen for indicated time. ULK1 proteins were immunoprecipitated, and ULK1 activity was measured. Data represent the mean ± SD from 3 independent experiments. *P*-values are from the two-sided t-tests. Bonferroni correction was used for multiple hypothesis correction.

(b) Indicated cells were incubated under 1% oxygen for 12 h. A streptavidin pulldown was performed. Exo, exogenous; Endo, endogenous.

(c) Indicated cells were incubated under 1% oxygen for 12 h. Atg14L precipitates were prepared using Ni-NTA beads, and VPS34 activity in the precipitates were measured. Exo, exogenous; Endo, endogenous. Data represent the mean ± SD from 3 independent experiments. *P*-values are from the two-sided t-tests.

(d) Alignment analysis of protein sequences spanning ULK1 R170 and T180 among species.

(e) LN229 cells were incubated under 1% oxygen for 12 h in the presence or absence of ULK1 pT180 peptide.

(f) LN229 cells were stimulated with hypoxia condition (1% oxygen) for 12 h, glucose deprivation for 2 h or amino acid deprivation for 4 h.

(g) Indicated LN229 cells were incubated under 1% oxygen for 12 h. A streptavidin pulldown assay was performed. Exo, exogenous; Endo, endogenous.

(h) Endogenous ULK1-depleted LN229 cells with reconstituted expression of WT SFB-ULK1, SFB-ULK1 T180A or exogenous expression of His-Atg14L were incubated under 1% oxygen for 12 h. Atg14L precipitates were prepared, and the VPS34 activity in the precipitates were measured. Exo, exogenous; Endo, endogenous. Data represent the mean ± SD from 3 independent experiments. *P*-value is from the two-sided t-test.

(i) Endogenous ULK1-depleted LN229 cells with reconstituted expression of WT SFB-ULK1, SFB-ULK1 T180A or exogenous expression of LC3-GFP were incubated under 1% oxygen for 12 h. Formation of LC3 puncta was analyzed. Scale bar, 10 μm. Data represent the mean ± SD from 3 independent experiments. *P*-value is from the two-sided t-test.

Source data are provided as a Source Data file.

Supplementary Figure 5

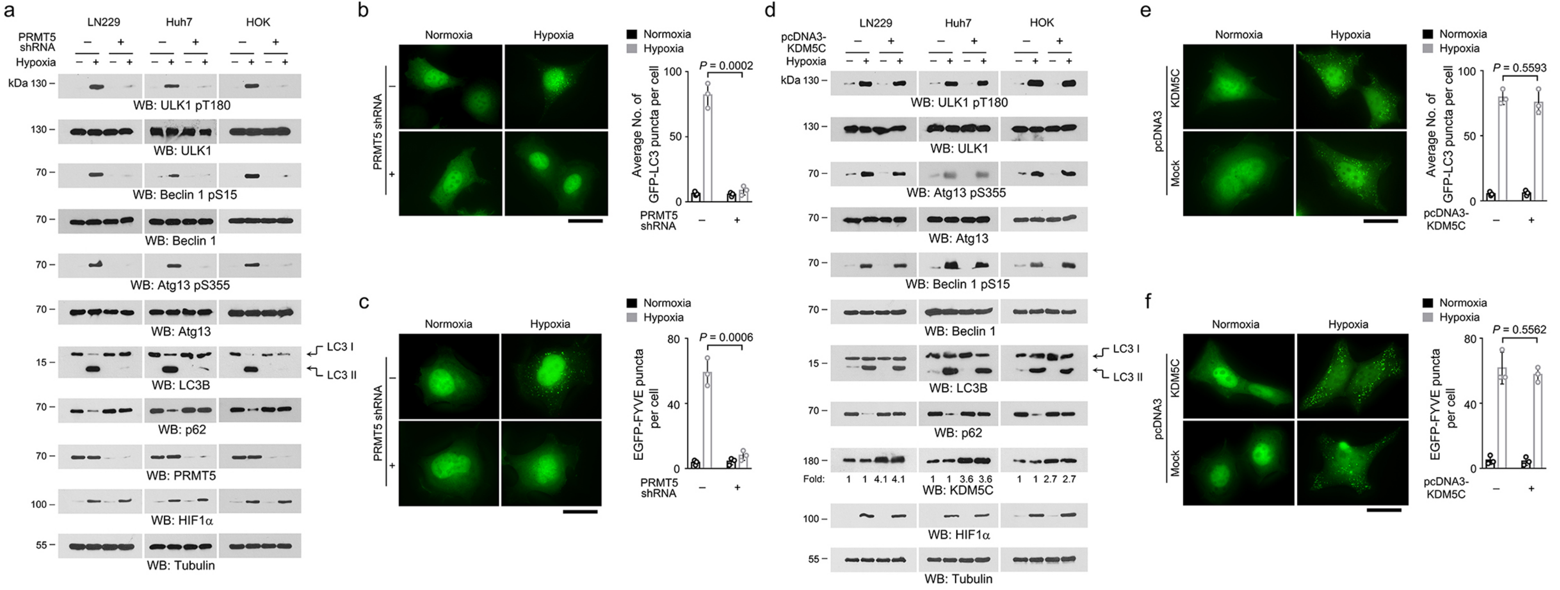

Supplementary Figure 5 PRMT5 expression affects hypoxia-induced ULK1 autophagy

(a) LN229, Huh7 and HOK cells were transfected with PRMT5 shRNA. 48 h after transfection, cells were incubated under 1% oxygen for 12 h. Immunoblot with indicated antibodies were performed.

(b-c) PRMT5-depleted LN229 cells were transfected with GFP-LC3 (b) or EGFP-FYVE (c) plasmid. 48 h after transfection, cells were incubated under 1% oxygen for 12 h, and fluorescent puncta were analyzed. Data represent the mean ± SD from 3 independent experiments. *P*-values are from the two-sided t-tests. Scale bar, 10 μm (b); Scale bar, 7 μm (c).

(d) LN229, Huh7 and HOK cells were transfected with pcDNA3-KDM5C. 48 h after transfection, cells were incubated under 1% oxygen for 12 h. Immunoblot with indicated antibodies were performed.

(e-f) KDM5C-overexpressed LN229 cells were transfected with GFP-LC3 (b) or EGFP-FYVE (c) plasmid. 48 h after transfection, cells were incubated under 1% oxygen for 12 h, and fluorescent puncta were analyzed. Data represent the mean ± SD from 3 independent experiments. *P*-values are from the two-sided t-tests. Scale bar, 10 μm (e); Scale bar, 8 μm (f).

Source data are provided as a Source Data file.

Supplementary Figure 6

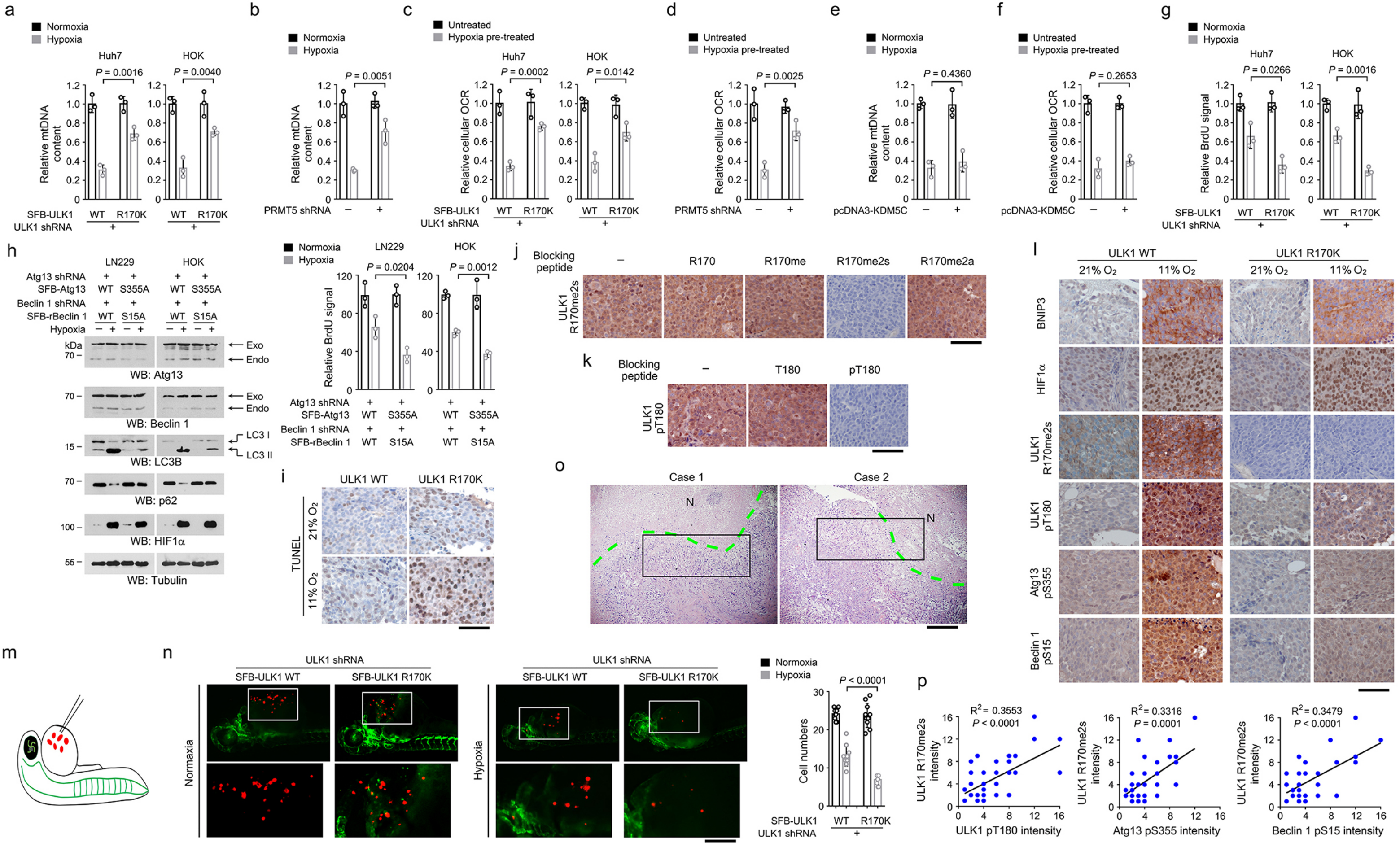

Supplementary Figure 6 ULK1 R170me2s promotes mitochondria turnover and rescues tumor growth in response to hypoxia

(a-g) Data was normalized to the WT untreated group.

(a, c, g, h) Indicated cells were incubated under 1% oxygen for 24 h (a), 18 h (c), 48 h (g), or 60 (h). The level of mitochondrial DNA (a), the cellular OCR in normoxia condition (c) or BrdU incorporation (g, h) was measured. Data represent the mean ± SD from 3 independent experiments. *P*-values are from the two-sided t-tests. r, shRNA-resistant.

(b, d, e, f) LN229 cells with expression of PRMT5 shRNA (b, d) or pcDNA3-KDM5C plasmid (e, f) were incubated under 1% oxygen for 24 h (b, e) or 18 h (d, f). The level of mitochondrial DNA (b, e) and cellular OCR in normoxia condition (d, f) was measured. Data represent the mean ± SD from 3 independent experiments. *P*-values are from the two-sided t-tests.

(i) TUNEL analyses of mice tumor tissue in Figure 4g. Scale bar, 75 μm.

(j-k) Immunochemical staining using anti-ULK1 R170Kme2s (j) or anti-ULK1 pT180 (k) antibody in presence of indicated blocking peptide. Scale bar, 75 μm.

(l) Immunochemical staining of mouse brain tumor tissues in Figure 4g. Scale bar, 50 μm.

(m) Schematic of tumor cells injection in zebrafish embryos.

(n) Indicated HOK cells stained with CM-Dil Dye (red) were microinjected into the pericardial cavity of Tg(flk1: EGFP) zebrafish (blood vessels shown in green). The zebrafish were maintained in normoxia or hypoxia condition for 5 days, and the number of dye-labeled cells in each group (n = 10) were counted. The boxed areas (scale bar, 250 μm) are enlarged (scale bar, 100 μm) and shown underneath. Data represent the mean ± SD. *P*-value is from the two-sided t-test.

(o) H&E staining of sample Case1 and Case 2 in Figure 4i. The boxed areas were shown in Figure 4i. The border of necrotic regions was indicated with green dotted line. N, necrotic region.

(p) Quantification of the immunochemical staining in Figure 4i (n = 40).

Source data are provided as a Source Data file.

Supplementary Table 1 The sequences used for primers

Name	Sequence
D-Loop forward	GAT TTG GGT ACC ACC CAA GTA TTG
D-Loop reverse	GTA CAA TAT TCA TGG TGG CTG GCA
β-Actin forward	TCA CCC ACA CTG TGC CCA TCT ACG A
β-Actin reverse	CAG CGG AAC CGC TCA TTG CCA ATG G
