# Supplementary material for: Oxygen-sensitive methylation of ULK1 is required for hypoxia-induced autophagy
Source: Nat Commun. 2022 Mar 4;13:1172. doi: 10.1038/s41467-022-28831-6 (PMC8897422; doi:10.1038/s41467-022-28831-6)
Supplement: Supplementary file 2 — Reporting Summary [file 41467_2022_28831_MOESM2_ESM.pdf]

nature research

Corresponding author(s): RUI LIU

Last updated by author(s): Jan 27, 2022

Reporting Summary

Nature Research wishes to improve the reproducibility of the work that we publish. This form provides structure for consistency and transparency in reporting. For further information on Nature Research policies, see Authors & Referees and the Editorial Policy Checklist.

Statistics

For all statistical analyses, confirm that the following items are present in the figure legend, table legend, main text, or Methods section.

n/a	Confirmed	
☐	☒	The exact sample size (*n*) for each experimental group/condition, given as a discrete number and unit of measurement
☐	☒	A statement on whether measurements were taken from distinct samples or whether the same sample was measured repeatedly
☐	☒	The statistical test(s) used AND whether they are one- or two-sided<br>*Only common tests should be described solely by name; describe more complex techniques in the Methods section.*
☒	☐	A description of all covariates tested
☒	☐	A description of any assumptions or corrections, such as tests of normality and adjustment for multiple comparisons
☐	☒	A full description of the statistical parameters including central tendency (e.g. means) or other basic estimates (e.g. regression coefficient) AND variation (e.g. standard deviation) or associated estimates of uncertainty (e.g. confidence intervals)
☐	☒	For null hypothesis testing, the test statistic (e.g. *F*, *t*, *r*) with confidence intervals, effect sizes, degrees of freedom and *P* value noted<br>*Give P values as exact values whenever suitable.*
☒	☐	For Bayesian analysis, information on the choice of priors and Markov chain Monte Carlo settings
☒	☐	For hierarchical and complex designs, identification of the appropriate level for tests and full reporting of outcomes
☒	☐	Estimates of effect sizes (e.g. Cohen's *d*, Pearson's *r*), indicating how they were calculated

Our web collection on statistics for biologists contains articles on many of the points above.

Software and code

Policy information about availability of computer code

Data collection	n/a
Data analysis	GraphPad Prism 7 software was used for statistical analyses. Fiji (ImageJ2) software package was used for cell counting for zebrafish experiments. Proteome Discoverer software program (version 1.4; Thermo Fisher Scientific) was used for the proteomic analyses.

For manuscripts utilizing custom algorithms or software that are central to the research but not yet described in published literature, software must be made available to editors/reviewers. We strongly encourage code deposition in a community repository (e.g. GitHub). See the Nature Research guidelines for submitting code & software for further information.

Data

Policy information about availability of data

All manuscripts must include a data availability statement. This statement should provide the following information, where applicable:

- Accession codes, unique identifiers, or web links for publicly available datasets
- A list of figures that have associated raw data
- A description of any restrictions on data availability

All the data supporting the findings of this study are available within the article and its supplementary information files. The proteomic data have been deposited in the Mendeley Data (doi: 10.17632/rvxrpbxsmz.1; https://data.mendeley.com/datasets/rvxrpbxsmz/1) without accession codes. Source data are provided with this paper. All relevant data are available upon reasonable request.

Field-specific reporting

Please select the one below that is the best fit for your research. If you are not sure, read the appropriate sections before making your selection.

☒ Life sciences ☐ Behavioural & social sciences ☐ Ecological, evolutionary & environmental sciences

For a reference copy of the document with all sections, see nature.com/documents/nr-reporting-summary-flat.pdf

Life sciences study design

All studies must disclose on these points even when the disclosure is negative.

Sample size	For animal experiments, the rationale for analyzing multiple cells/fields from 8-10 biologically independent mice in each group (exact numbers mentioned in figure legends) was based on a resource efficient strategy common across relevant publications (Jiang et al. 2020 Nat Commun, PMID: 33046710; Harney et al. 2015 Cancer Disc, PMID: 26269515; Karagiannis et al. 2017 Sci Transl Med, PMID: 28679654). For all other experiments, cells/fields measurement were made in at least 3 biological replicates and is also common across relevant publications (Jiang et al. 2020 Nat Commun, PMID: 33046710; Pignatelli et al. 2016 Sci Rep, PMID: 27901093; Cabrera et al. 2018 Breast Cancer Res, PMID: 29636067; Tao et al. 2020 J Exp Clin Cancer Res, PMID: 32943090).
Data exclusions	No samples or animals were excluded from the analyses.
Replication	All experiments were repeated independently with similar results for at least three times.
Randomization	Randomization of samples were not applicable in this paper
Blinding	The investigators who performed quantification of immunostaining were blinded to the patient information. Blinding is not applicable for all other experiments in this paper.

Reporting for specific materials, systems and methods

We require information from authors about some types of materials, experimental systems and methods used in many studies. Here, indicate whether each material, system or method listed is relevant to your study. If you are not sure if a list item applies to your research, read the appropriate section before selecting a response.

Materials & experimental systems

n/a	Involved in the study	
☐	☒	Antibodies
☐	☒	Eukaryotic cell lines
☒	☐	Palaeontology
☐	☒	Animals and other organisms
☒	☐	Human research participants
☒	☐	Clinical data

Methods

n/a	Involved in the study	
☒	☐	ChIP-seq
☒	☐	Flow cytometry
☒	☐	MRI-based neuroimaging

Antibodies

Antibodies used

Anti-ULK1 (Rabbit), 1: 1000, Cell Signaling Technology, #8054, D8H5
Anti-HIF1α (Rabbit), 1:1000, Cell Signaling Technology, #36169, D1S7W
Anti-α-Tubulin (Rabbit), 1:2000, Cell Signaling Technology, #2125, 11H10
Anti-LC3B (Mouse), 1:1000, Cell Signaling Technology, #83506, E5Q2K
Anti-SQSTM1/p62 (Mouse), 1:1000, Cell Signaling Technology, #88588, D5L7G
Anti-Beclin 1 (Mouse), 1:1000, Cell Signaling Technology, #4122,2A4
Anti-Beclin 1 pS15 (Rabbit), 1:500, Cell Signaling Technology, #84966, D4B7R
Anti-Atg13 (Rabbit), 1:1000, Cell Signaling Technology, #13468, E1Y9V
Anti-Atg13 pS355 (Rabbit), 1:500, Cell Signaling Technology, #46329, E4D3T
Anti-S6K pT421/pS424 (Rabbit), 1:1000, Cell Signaling Technology, #9204,
Anti-Tom20 (Rabbit), 1:500, Cell Signaling Technology, #42406, D8T4N
Anti-Hamartin/TSC1 (Rabbit), 1:500, Cell Signaling Technology, #6935, D43E2
Anti-AMPKα (Rabbit), 1:1000, Cell Signaling Technology, #5832, D63G4
Anti-ULK2 (Rabbit), 1:1000, Abcam, ab97695,
Anti-PRMT5 (Rabbit),1:1000, Abcam, ab109451, EPR5772
Anti-KDM5C (Rabbit),1:1000, Abcam, ab194288, EPR18653
Anti-BNIP3 (Rabbit), 1:1000, Abcam, ab109362, EPR4034
Anti-S6K1 (Rabbit), 1:1000, Abcam, ab32529,E343
Anti-ACC (Rabbit), 1:1000, Abcam, ab109368, EPR4971
Anti-ACC pS79 (Rabbit), 1:500, Abcam, ab68191, EP1885Y

	Anti-ULK1 pS757 (Rabbit), 1:500, Abcam, ab229909, EPR22265-9<br>Anti-ULK1 pS555 (Rabbit), 1:500, Abcam, ab229537,<br>Anti-Flag (Rabbit), 1:5000, Sigma, F2555<br>Anti-KDM4E (Rabbit), 1:1000, Merck, ABE1081<br>Rabbit polyclonal antibody recognizing phosphorylated ULK1 pT180 (1: 1000), ULK1 R170me2s (1: 1000) and ULK1 R170me2a (1: 1000) were customized from Boer Biotechnology (Chengdu, China).
Validation	All the antibodies used in this work have been validated by the companies where these antibodies were purchased from, by western blot using human and mouse cell lysate samples.

Eukaryotic cell lines

Policy information about cell lines

Cell line source(s)	LN229 GBM cells and 293T cells were obtained from ATCC. Huh7 HCC cells were obtained from JCRB Cell Bank. HOK cells were obtained from ScienCell.
Authentication	Short tandem repeat profiling was used for authentication
Mycoplasma contamination	All cells lines are confirmed without Mycoplasma contamination.
Commonly misidentified lines<br>(See ICLAC register)	No commonly misidentified lines were used

Animals and other organisms

Policy information about studies involving animals; ARRIVE guidelines recommended for reporting animal research

Laboratory animals	4-week-old female athymic nude BALB/c mice were used in this study. Mice were housed under ambient temperature of 24 ± 2° C, circulating air, constant humidity of 50 ± 10% and a 12 h :12 h light/dark cycle.<br>Zebrafish xenograft was established using embryos of Tg(flk1: EGFP) zebrafish strain obtained 48 hours postfertilization. Zebrafish were maintained under temperature at 28°C, pH 7.2–7.4, and a 14 h-on/10 h-off light cycle.
Wild animals	No wild animals were used in the study.
Field-collected samples	No field collected samples were used in the study
Ethics oversight	The use of animals was approved by the institutional review board of Chengdu Medical College.

Note that full information on the approval of the study protocol must also be provided in the manuscript.
